# β-Conglutins’ Unique Mobile Arm Is a Key Structural Domain Involved in Molecular Nutraceutical Properties of Narrow-Leafed Lupin (*Lupinus angustifolius* L.)

**DOI:** 10.3390/ijms24087676

**Published:** 2023-04-21

**Authors:** Elena Lima-Cabello, Julia Escudero-Feliu, Andreina Peralta-Leal, Pedro Garcia-Fernandez, Kadambot H. M. Siddique, Karam B. Singh, Maria I. Núñez, Josefa León, Jose C. Jimenez-Lopez

**Affiliations:** 1 Spanish National Research Council (CSIC), Estacion Experimental del Zaidin, Department of Stress, Development and Signaling in Plants, E-18008 Granada, Spain; 2 Biosanitary Research Institute of Granada (ibs. GRANADA), E-18012 Granada, Spain; 3 Research Centre for Information and Communications Technologies (CITIC-UGR), University of Granada, E-18071 Granada, Spain; 4 The UWA Institute of Agriculture, The University of Western Australia, Perth, WA 6001, Australia; 5 CSIRO Agriculture and Food, Floreat, WA 6014, Australia; 6 Centre for Crop and Disease Management, Curtin University, Bentley, WA 6102, Australia; 7 Biopathology and Regenerative Medicine Institute (IBIMER), University of Granada, E-18100 Granada, Spain; 8 Department of Radiology and Physical Medicine, Faculty of Medicine, University of Granada, E-18016 Granada, Spain; 9 Clinical Management Unit of Digestive Disease and UNAI, San Cecilio University Hospital, E-18006 Granada, Spain

**Keywords:** legumes, sweet lupin, vicilin, molecular nutraceutics, anti-inflammatory, redox regulatory capacity, mobile arm structural domain, truncated β-conglutins

## Abstract

Narrow-leafed lupin (NLL; *Lupinus angustifolius* L.) has multiple nutraceutical properties that may result from unique structural features of β-conglutin proteins, such as the mobile arm at the N-terminal, a structural domain rich in α-helices. A similar domain has not been found in other vicilin proteins of legume species. We used affinity chromatography to purify recombinant complete and truncated (without the mobile arm domain, tβ5 and tβ7) forms of NLL β5 and β7 conglutin proteins. We then used biochemical and molecular biology techniques in ex vivo and in vitro systems to evaluate their anti-inflammatory activity and antioxidant capacity. The complete β5 and β7 conglutin proteins decreased pro-inflammatory mediator levels (e.g., nitric oxide), mRNA expression levels (iNOS, TNFα, IL-1β), and the protein levels of pro-inflammatory cytokine TNF-α, interleukins (IL-1β, IL-2, IL-6, IL-8, IL-12, IL-17, IL-27), and other mediators (INFγ, MOP, S-TNF-R1/-R2, and TWEAK), and exerted a regulatory oxidative balance effect in cells as demonstrated in glutathione, catalase, and superoxide dismutase assays. The truncated tβ5 and tβ7 conglutin proteins did not have these molecular effects. These results suggest that β5 and β7 conglutins have potential as functional food components due to their anti-inflammatory and oxidative cell state regulatory properties, and that the mobile arm of NLL β-conglutin proteins is a key domain in the development of nutraceutical properties, making NLL β5 and β7 excellent innovative candidates as functional foods.

## 1. Introduction

The bioactive compounds in many legume seeds have been identified and characterized as functional food sources [1]. Interestingly, legume seed proteins have multiple benefits in the human diet due to their nutritional [2] and nutraceutical [3] properties. As a result, legume seed production and consumption have increased globally in recent decades [4].

Legume proteins exhibit antioxidant and anti-inflammatory properties and extend the amelioration capability of inflammatory-derived diseases [5]. For example, soybean lectin-like proteins have modulatory properties as anti-inflammatory molecules when present in circulating blood because they promote an inhibitory effect on neutrophil migration to the tissues, avoiding the enhancement of the inflammatory process [6]. 2S albumin has anti-inflammatory properties associated with nuclear factor kappa-light-chain-enhancer of activated B cells (NFκB) functional pro-inflammatory signal pathway suppression, since NFκB is responsible for activating the expression of pro-inflammatory genes including cytokines, chemokines, and adhesion molecules [7], and phospholipase A2 and cyclooxygenase-2 enzyme inhibition [8]. Bioactive peptides produced in germinating soybean seeds inhibited the synthesis of pro-inflammatory molecules, i.e. nitric oxide (NO), tumor necrosis factor alpha (TNFα), and prostaglandin E2 (PGE2) and expression of inducible nitric oxide synthase (iNOS) and cyclooxygenase-2 (COX-2) in in vitro experiments using LPS-stimulated macrophages [9]. The peptides from germinated chickpea protein suppressed NO synthesis in the lower gastrointestinal tract, suggesting they prevent inflammatory bowel diseases [10]. Pea albumin plays a protective role in colitis, reducing inflammatory parameters by inhibiting pro-inflammatory gene expression and decreasing cytokine release [11].

Other studies in the last decade have shown that sweet lupin species (*Lupinus albus*, *L. angustifolius*, *L. luteus*, and *L. mutabilis*) have anti-inflammatory properties [12], and extend anti-diabetic [13,14], antioxidant [15], and anti-cholesterolemic [16] properties in seed protein fractions. These studies refer to β- [17] and γ-conglutins. γ-conglutin proteins have been studied broadly for their glycaemia-regulating activity using different approaches and models [18], in vitro (INS-1E cells), ex vivo (human intestinal epithelial Caco-2 cells, and porcine IPEC-J2 cells) [19], and in vivo (male Wistar rats) [20]. The multi-functional nature of β-conglutin proteins sourced from narrow-leafed lupin (NLL, *L. angustifolius*) is considered the primary contributor to their varied nutraceutical properties [21,22]. Seven functional genes of β-conglutins in *L. angustifolius* conform proteins of about 650 amino acids length, ranging from 65–72 kDa. The structure of these β-conglutin isoforms comprise a globular domain and an N-terminal mobile arm exhibiting a noticeable polymorph [17]. 

Recent studies have shed light on the cytotoxic capacity of specific NLL β-conglutin protein isoforms (mainly β1) on breast cancer cells and their ability to act as sensitizers for drugs and treatments due to their target effect on stem cells. These β-conglutin proteins help prevent the malignant transformation of healthy cells, reducing metastasis and recurrence in breast cancer cell lines [23]. 

This study characterizes the anti-inflammatory properties of NLL conglutin β5 and β7 isoforms as new nutraceutical proteins and assesses their N-terminal domain as the underlying key structural feature promoting nutraceutical properties.

## 2. Results and Discussion

### 2.1. Purification and Identification of tβ5 and tβ7

**Figure 1:**
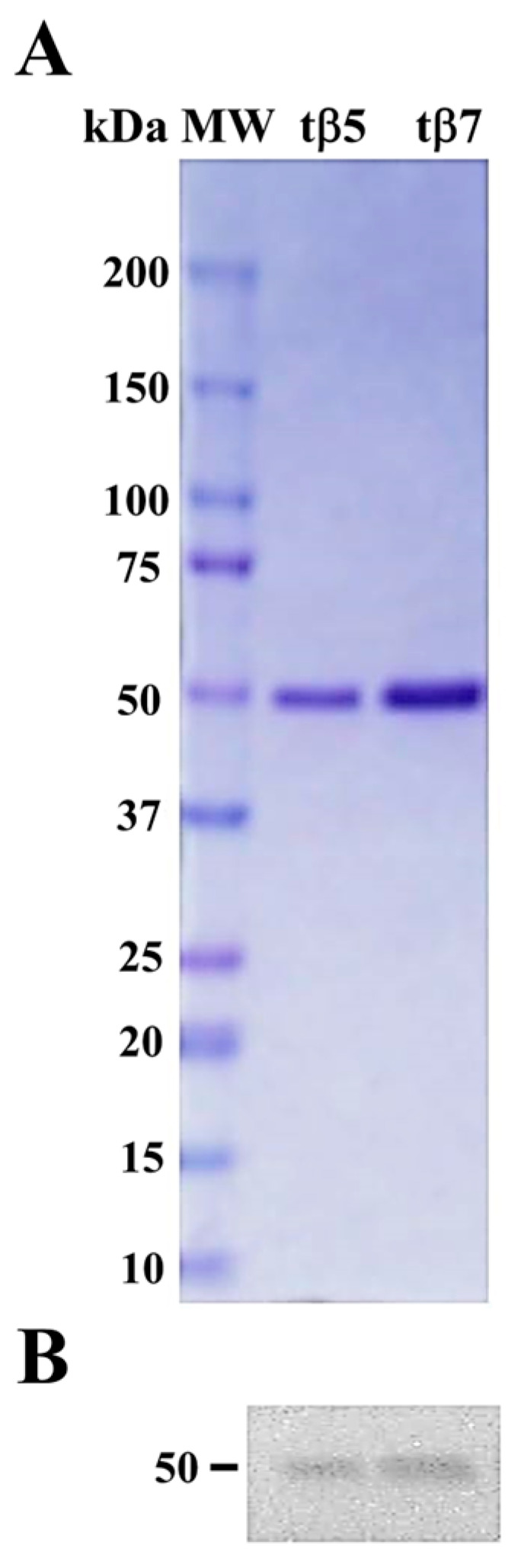
**Figure 1**. Purification and identification of tβ5 and tβ7 conglutin proteins. Two purified conglutin tβ5 and tβ7 proteins stained with Coomassie Brilliant Blue had a high level of purity (>95%) (**A**); Immunoblotting identified the same two purified β-conglutin proteins as the anti-β-conglutin antibody. MW, molecular weight standard (kDa) (**B**).

β-conglutin protein isoforms were expressed following the induction protocol by Jimenez-Lopez et al. 2016 [22] with modifications. Protein-purified chromatographic fractions were analyzed using SDS–PAGE to identify those containing recombinant proteins. The SDS–PAGE analyses of the eluted fractions showed a single protein band of approximately 50 kDa (Figure 1A), where recombinant β-conglutins exhibited >95% purity and 17–35 mg/mL. Immunoblotting with the anti-β-conglutin protein antibody confirmed the identity of β-conglutin protein isoforms (Figure 1B).

### 2.2. β-Conglutin Proteins Structural Modeling

An in silico structural analysis of seed β-conglutins identified a new family of major allergen proteins in lupin, which was compared to other relevant food allergens, such as Ara h 1 [24]. Residue variability in IgE-binding epitopes might be a major contributor to the observed differences in cross-reactivity among legumes [25].

Structural analysis of the β5 and β7 conglutin isoforms was performed with I-Tasser (fold recognition) using protein templates from the PDB library. Models were validated and conservation analyzed through different structural parameters. Three-dimensional structures of truncated β5 and β7 conglutins were illustrated in a cartoon diagram, with α-helices, β-sheets, and coils depicted in red, yellow, and green, respectively, integrating the main protein domains (Figure S1).

β-conglutins have distinct structural features, including two cupin domains forming a Rossmann-fold-like structure found in many enzymes using molecular oxygen as a substrate, shaping a globular protein domain [26]. This domain comprises a negatively charged surface integrated by two conserved β-barrels, each comprising antiparallel β-sheets that form an extended beta-sheet with relatively large polymorphisms (Figure S2). A mobile N-terminal arm comprises 8–10 α-helices with large polymorphisms in their sequences (Figure S2). This arm is a distinct feature in NLL β-conglutin proteins compared to other proteins of the vicilin family among legume species [24,25]. 

No other vicilin-like proteins similar to β-conglutins with this unique mobile arm structure have been characterized. A very small N-terminal sequence has been described in the phaseolin protein (PDB: 1PHS) from *P. vulgaris* [27].

Molecular modeling analysis of β5 and β7 has revealed considerable structural differences between these and other NLL β-conglutin isoforms, particularly affecting 2D elements (loops and coils), with more noticeable differences in the N-terminal domain containing the mobile arm affecting number, length, amino acid composition, and sequence polymorphism (Figures S1 and S2). Thus, some putative functional differences among NLL β-conglutins could be due to these structural differences [17,24,25,26].

### 2.3. β-Conglutin Protein Inhibits the Gene Expression of Different Cytokines and Pro-Inflammatory Mediators

A recent study investigated the anti-inflammatory properties of NLL γ-conglutin proteins from mature seeds using in vitro human PANC-1 pancreatic cell lines in an induced inflammation model using bacteria lipopolysaccharide (LPS) [14]. An induced insulin-resistant (IR) cell model assessed the capability of NLL γ-conglutins to improve the oxidative stress homeostasis of cells, inflammatory induced state, and IR improvements with a plethora of functional effects at the molecular level by decreasing protein carbonylation, nitric oxide synthesis, and inducible nitric oxide synthase (iNOS) transcriptional levels, and increasing glutathione (GSH), catalase (CAT), and superoxide dismutase (SOD) activities, among others. These results indicate that NLL γ-conglutin proteins could be used in functional foods and implemented in alternative diagnoses and therapeutic molecular tools to prevent and treat inflammatory-related diseases [28].

**Figure 2:**
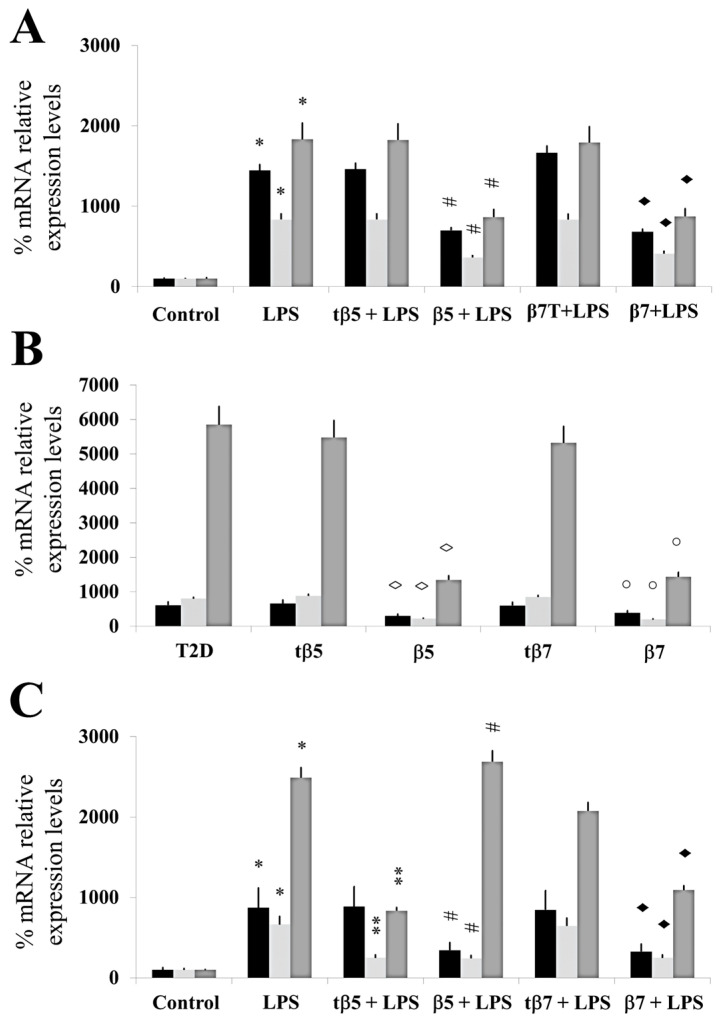
Assessment of mRNA expression levels of TNF-α, IL-1β, and iNOS genes. Culture cells of HepG2 (**A**), T2D (**B**), and healthy control subjects (**C**). Each group of cells was incubated for 24 h in LPS, LPS+tβ5, LPS+β5, LPS+tβ7, or LPS+β7. Bars show TNF-α (black), IL-1β (punted white), and iNOS (gray) color in each cell type described above. * *p* ˂ 0.05 LPS vs. control; ** *p* ˂ 0.05 LPS+tβ5 or tβ7 vs. LPS; # *p* ˂ 0.05 LPS+β5 vs. LPS; ◆ *p* ˂ 0.05 LPS+β7 vs. LPS. ◊ *p* ˂ 0.05 LPS+β5 vs. T2D, and ○ *p* ˂ 0.05 LPS+β7 vs. T2D in T2D cell cultures. Data represent the mean ± SD of three independent experiments.

We assessed the capacity of β5 and β7 conglutin proteins to reduce the gene expression of pro-inflammatory molecules, finding that truncated forms of NLL β5 and β7 conglutins do not promote these beneficial effects (Figure 2). 

For inflammatory response analysis, real-time quantitative PCR technology was used to assay TNF-α, IL-1β, and iNOS mRNA expression from each experimental group. Total RNA was extracted from HepG2 cells and isolated blood and PBMC cultures from type 2 diabetic (T2D) patients and control subjects using an RNeasy Mini Kit (Qiagen). 

LPS significantly induced an inflammatory state in HepG2 (* *p* ˂ 0.05 LPS vs. control) since relative % of mRNA expression increased by 1370, 845, and 1672, respectively, for TNFα, IL-1 β, and iNOS. tβ5 and tβ7 did not significantly inhibit the inflammatory state induced by LPS in HepG2 cells (Figure 2A). β5 and β7 significantly inhibited (down-regulated) the relative % of mRNA expression levels of pro-inflammatory mediators (TNFα, IL-1 β, and iNOS) increased by LPS by 35, 585, and 1076 (# *p* ˂ 0.05 LPS+β5 vs. LPS), and 745, 527, and 903 (◆ *p* ˂ 0.05 LPS+β7 vs. LPS) (Figure 2A), respectively.

The mRNA expression level of TNFα, IL-1β, and iNOS genes did not significantly differ in tβ5- and tβ7-challenged cell cultures.

Different diseases, such as T2D, obesity, and metabolic syndrome, are associated and chronically sustained inflammation. Depending on the genetic background and environment, multiple mechanisms could underlie the inflammatory response and contribute to the pathology [29]. 

The main contributors to inflammatory development are increased levels of different cytokines, NO, and iNOS mRNA expression. For instance, sustained exposure to IL-1β activates iNOS expression, resulting in excessive NO production that induces the expression of pro-inflammatory genes [30].

T2D is characterized by impaired insulin secretion and/or insulin sensitivity [31] and sustained by chronic subclinical inflammation. Recent results [13] have revealed the possibility of using particular β-conglutin proteins to prevent and treat diabetes and their potential as anti-inflammatory molecules. iNOS mRNA and protein levels substantially increased in T2D in all experimental groups compared to the control.

In the present study, the mRNA expression levels of TNF-α, IL-1β, and iNOS genes in T2D cells culture challenged with β5, β7, tβ5, or tβ7 followed a similar reduction pattern in expression level compared to the T2D group, decreasing to 310, 734, and 4157 for β5 (◊ *p* ˂ 0.05 β5 vs. T2D) and 365, 710, and 3470 for β7 (○ *p* ˂ 0.05 β7 vs. T2D), respectively. No significant (*p* > 0.05) reductions in mRNA expression levels of TNFα, IL-1β, and iNOS occurred with tβ5 or tβ7 (Figure 2B).

The results also showed that LPS significantly induced an inflammatory state (mRNA levels of TNFα, IL-1 β, and iNOS) in culture cells of healthy control subjects (* *p* ˂ 0.05 LPS vs. control), with no significant differences in culture cells challenged with tβ5 and tβ7.

Conglutin β5 significantly inhibited the induced inflammatory state by LPS in culture cells of healthy control subjects for mRNA expression levels of TNFα and IL-1β [540 and 465, respectively] compared to LPS-treated cells (#*p* ˂ 0.05 LPS+β5 vs. LPS), but not for iNOS mRNA expression levels (*p* < 0.05). β7 conglutin significantly inhibited the inflammatory state for mRNA expression levels of TNFα, IL-1 β, and iNOS (◆ *p* ˂ 0.05 LPS+β7 vs. LPS) by 560, 370, and 1420, respectively. These results indicate that β5 and β7 conglutin proteins reduce the pro-inflammatory capacity in healthy subjects’ culture cells by diminishing TNFα, IL-1β, and iNOS mRNA expression levels, thus promoting the amelioration of the inflammatory process (Figure 2C). However, no significant differences occurred when tβ5 or tβ7 challenged these cell cultures (*p* > 0.05).

### 2.4. β5 and β7 Conglutins Can Decrease the Production of Pro-Inflammatory Mediators

Cytokines are crucial in the functional regulation of inflammatory cell stages, where certain organs (e.g., pancreas) respond to physio-pathological situations by producing cytokines to regulate their own functions [32]. During the establishment and progression of T2D, pro-inflammatory cytokines are imbalanced, causing dysfunction in tissue cells [33]. In this regard, restoring normal cytokine levels may prevent and even restore the physiological state of cells, i.e., reversion of T2D progression [34]. 

**Figure 3:**
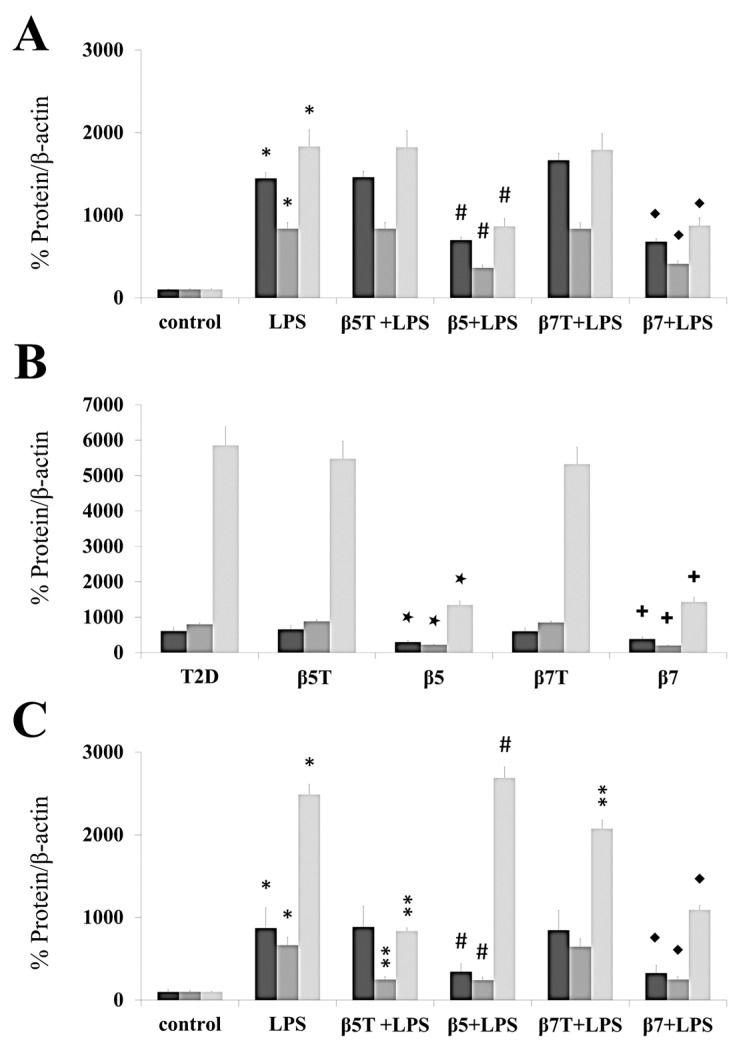
β5 and β7 conglutins reduced the protein levels of TNF-α, IL-1β, and iNOS. Culture cells of HepG2 (**A**), T2D (**B**), and healthy control subjects (**C**). Each group of cells was incubated for 24 h in the presence of LPS, LPS+tβ5, LPS+β5, LPS+tβ7, and LPS+β7. Bars show TNF-α (dark gray), IL-1β (gray), and iNOS (light gray) for each cell type described above. * *p* ˂ 0.05 LPS vs. control; ** *p* ˂ 0.05 LPS+tβ5 or tβ7 vs. LPS; # *p* ˂ 0.05 LPS+β5 vs. LPS; ◆ *p* ˂ 0.05 LPS+β7 vs. LPS. ★ *p* ˂ 0.05 β5 vs. T2D, and **+** *p* ˂ 0.05 β7 vs. T2D in T2D cell cultures. Data represent the mean ± SD of three independent experiments.

In this study, we used the ELISA method to assess the anti-inflammatory properties of β5, β7, tβ5, and tβ7 conglutin proteins through their capacity to modify the amount of important pro-inflammatory mediators, such as TNF-α, INFγ, MPO, S-TNF-R1, S-TNF-R2, TWEAK, and cytokines (IL-1β, IL-2, IL-6, IL-8, IL-12, IL-17, and IL-27) (Figure 3, Table 1). The conglutin isoform/form challenges were made in the HepG2 cell model, with PMBC cells isolated from T2D and healthy subjects. The levels of the above pro-inflammatory mediators and cytokines were assessed under basal conditions, after cell treatment with LPS, and by challenging the cell culture individually with β5 and β7 (complete or truncated forms). The protein levels of TNF-α, INFγ, MPO, S-TNF-R1, S-TNF-R2, TWEAK, and cytokines (IL-1β, IL-2, IL-6, IL-8, IL-12, IL-17, and IL-27) significantly (*p* < 0.05) increased (several-fold) after the LPS challenges in these three cell cultures (Figure 3, Table 1). LPS+β-conglutin protein (β5 or β7, respectively) challenges significantly reduced the above pro-inflammatory mediators and cytokines, whereas the truncated forms tβ5 or tβ7 had no significant effect (Figure 3, Table 1).

These results agree with those in the RT-qPCR experiments (Figure 2), where all cell cultures had reduced mRNA and protein levels for the different pro-inflammatory mediators (TNFα, IL-1β, and iNOS) after challenging with the complete forms of β5 and β7, but no significant differences (*p* > 0.05) with the truncated forms, relative to the LPS treatment (Figure 3).

Few studies have shown anti-inflammatory effects of plant peptides for regulating the balance of pro-inflammatory mediators, such as INFγ, TNFα, NO, and cytokine production [35]. In this study, complete forms of NLL β5 and β7 conglutin proteins suppressed gene expression and protein production of pro-inflammatory molecules, indicating their potential as anti-inflammatory molecules that could help manage the detrimental effects of chronic inflammatory-based diseases, such as T2D [12,13,14]. Thus, the individual or synergistic action of combining these pro-inflammatory molecules can exert multiple deleterious effects on cells. For example, (i) sustained IL-1β, IL-6, IL-8, and IL-17 production due to high carbohydrate or lipid levels promotes excessive production and release of ROS, misbalancing the redox state. IL-17 has pleiotropic functional effects, including promoting the synthesis of TNFα and IL-6 on diverse cells [36]; therefore, reducing the IL-17 protein level (as β5 and β7 did) could reduce the pro-inflammatory effects of these cytokines; (ii) synergistic action of IL-1β+INFγ and even IL-1β+INFγ+TNFα cytokines increased NO production due to positive feedback on the iNOS gene, increasing inflammatory and apoptotic actions [32,37]. These effects were reverted by NLL β5 and β7 actions at the molecular level; (iii) IL-12 expression increased with the effect of INFγ, stimulating positive feedback for increasing INFγ synthesis [38]. Complete forms of NLL β5 and β7 molecular actions ameliorated these adverse inflammatory effects, while truncated NLL β5 and β7 protein forms did not.

The cytokine tumor necrosis factor-like weak inducer of apoptosis (TWEAK) is a member of the TNF receptor (TNFR) superfamily, and S-TNF-R1/-R2 can induce multiple biological activities, such as promoting angiogenesis, inflammatory cytokine synthesis, and apoptosis [39]. Myeloperoxidase (MPO) is a member of the heme-peroxidase family, which includes a set of enzymes with potent oxidoreductase activity. MPO and its oxidative products react with various lipids, proteins, and nucleic acids, causing detrimental effects in tissues associated with ongoing inflammatory states [40]. In this regard, this study showed that β5 and β7 decreased the protein levels of these pro-inflammatory modulators, but tβ5 and tβ7 did not.

### 2.5. Assessing the Antioxidant Regulatory Capacity of β5 and β7 Conglutins

Eliminating ROS molecules from cells strongly depends on enzymatic activities such as Cu/Zn-SOD, CAT, and GSH, crucial indicators of cellular antioxidant capacity and oxidative stress [41].

The results highlight the potential of β-conglutins (β5 and β7) for decreasing the pro-inflammatory capacity in HepG2, T2D, and healthy control cells by decreasing gene expression levels of TNFα, IL-1β, and iNOS, thus supporting inflammatory amelioration at the molecular level. The body contains a complex antioxidant defense grid that relies on endogenous enzymatic and non-enzymatic antioxidants to resist the damaging effects on vital biomolecules and body tissues. Based on their response to free radical invasion, antioxidants can be categorized as the first, second, third, and even fourth line of defense. The role and effectiveness of first-line defense antioxidants (e.g., SOD, CAT, and GPX) are important and indispensable for the entire defense strategy of antioxidants [42].

Recent studies have revealed that NLL γ-conglutin has a plethora of functional effects related to reducing cell oxidative stress induced by inflammation by decreasing protein carbonylation, nitric oxide synthesis, and inducible nitric oxide synthase (iNOS) transcriptional levels, increasing glutathione (GSH) levels, and modulating SOD and CAT activities [43]. High GSH and low SOD and CAT activities might be regulated by γ-conglutin protein through direct or indirect effects in avoiding lipid and protein oxidative modifications, supported by the concomitant reduction in oxidative carbonylation and overall improved oxidative stress balance, helping to reach an ameliorated inflammation molecular cellular statement by γ-conglutin protein as an antioxidant protein [14].

Interestingly, the lowered cellular pro-inflammatory capacity could be partly the result of the antioxidant capacity of β-conglutin, with changes in GSH levels and SOD and CAT enzymatic activities helping to keep redox homeostasis in T2D and other inflammatory-dependent diseases also affected by oxidative stress [12,13].

The present study treated cell cultures individually with β5, β7, tβ5, and tβ7 and measured SOD and CAT activities and GSH levels. Data were statistically analyzed.

**Figure 4:**
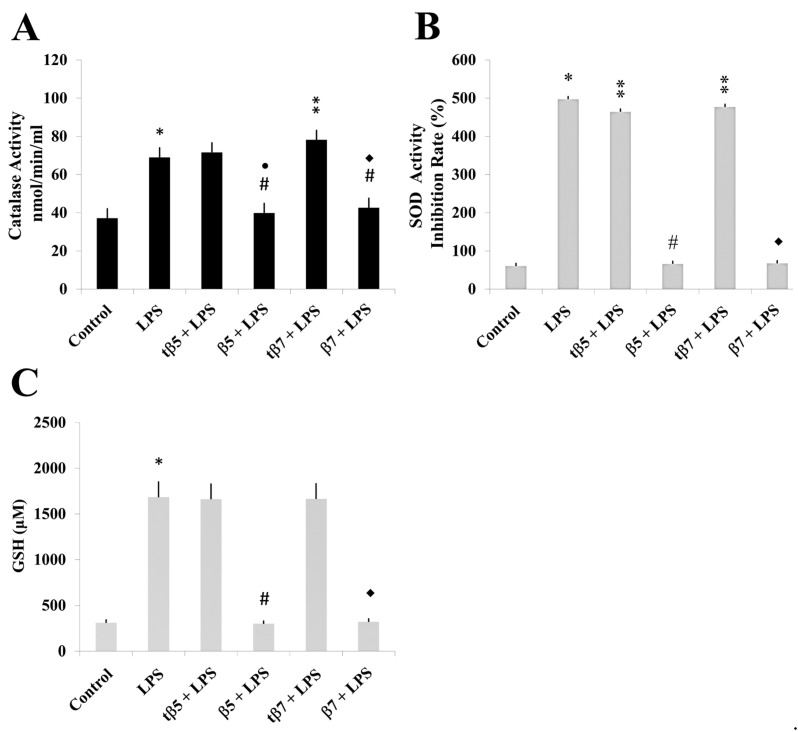
Effect of NLL β5 and β7 conglutins on CAT and SOD enzymatic activities and GSH production in HepG2 culture cells. CAT (**A**) and SOD (**B**) activities and GSH production (**C**). * *p* ˂ 0.05 LPS vs. control; ** *p* ˂ 0.05 LPS+tβ5 or LPS+tβ7 vs. LPS; # or ●# *p* ˂ 0.05 LPS+β5 vs. LPS; ◆ or ◆# *p* ˂ 0.05 LPS+β7 vs. LPS. Data represent mean ± SD from three independent experiments.

**Figure 5:**
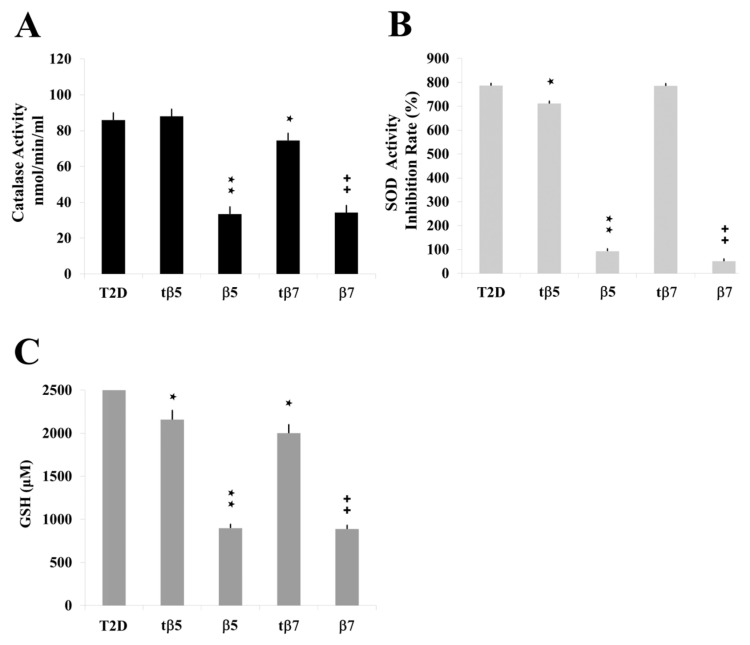
Effect of NLL β5 and β7 conglutins on CAT and SOD activities and GSH production in Type II diabetes (T2D) culture cells. CAT (**A**) and SOD (**B**) activities and GSH production (**C**). **★** *p* ˂ 0.05 T2D vs. tβ5 or tβ7; **★★** *p* ˂ 0.05 β5 vs. T2D; ++ *p* ˂ 0.05 β7 vs. T2D. Data represent mean ± SD from three independent experiments.

**Figure 6:**
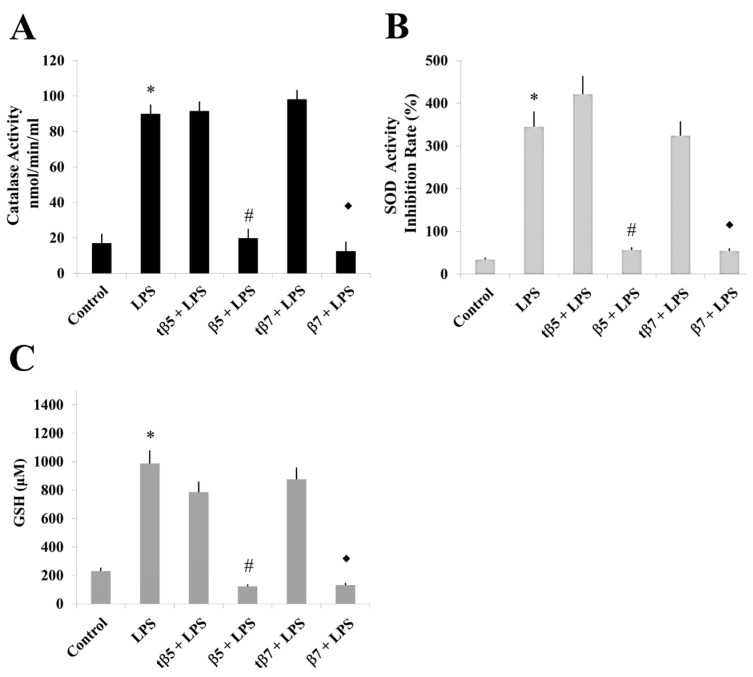
Effect of NLL β5 and β7 conglutins on CAT and SOD activities and GSH production in healthy subject culture cells. CAT (**A**) and SOD (**B**) activities and GSH production (**C**). * *p* ˂ 0.05 LPS vs. control; # *p* ˂ 0.05 LPS+β5 vs. LPS; ◆ *p* ˂ 0.05 LPS+β7 vs. LPS. Data represent mean ± SD from three independent experiments.

The LPS induced inflammatory states that significantly differed from control cell cultures (* *p* ˂ 0.05 LPS vs. control). The CAT activities of model culture cells (Figure 4), PMBC culture cells from T2D (Figure 5), and healthy subjects (Figure 6) decreased to 38 (Figure 4A), 32 (Figure 5A), and 20 (Figure 6A) nmol/min/mL, respectively, when challenged with β5 conglutin (* *p* ˂ 0.05 LPS vs. β5) and to 43 (Figure 4A), 31 (Figure 5A), and 17 (Figure 6A) nmol/min/mL, respectively, when challenged with β7 (*p ˂ 0.05 LPS vs. β7).

Similar results occurred for SOD activity and GSH levels measured in model culture cells (Figure 4), PMBC culture cells from T2D (Figure 5), and healthy subjects (Figure 6). The LPS-treated cells had higher SOD activities (Figure 4B, Figure 5B, and Figure 6B) and GSH levels (Figure 4C, Figure 5C, and Figure 6C) than control cell cultures (* *p* ˂ 0.05 LPS vs. control), while those challenged with β5 (# *p* ˂ 0.05 LPS+β5 vs. LPS) and β7 (◆ *p* ˂ 0.05 LPS+β7 vs. LPS) conglutins had significantly lower SOD activities (Figure 4B, Figure 5B, and Figure 6B) and GSH levels (Figure 4C, Figure 5C, and Figure 6C). No significant differences occurred for culture cells challenged with tβ5 or tβ7.

The behavior induced by NLL β5 and β7 conglutin proteins in the induced inflammatory stage of culture cells related to oxidative stress regulation (SOD and CAT activities, GSH levels) revealed that these conglutins can balance oxidative stress to improve the inflammatory stage of cells by regulating important antioxidant molecules. However, truncated conglutins (tβ5 and tβ7) do not promote these effects.

### 2.6. Effect of β5 and β7 Conglutins on NO Production

Nitric oxide is considered a key signaling molecule during inflammation [44]. Excessive ROS production, NO production by inducible NO synthase (iNOS), and expression of pro-inflammatory genes, such as TNF-α, IL-6, and IL-12, connect stress and inflammation in T2D patients [30]. Interestingly, excess NO production is detrimental to the host, leading to the development of inflammatory-related diseases [44].

In a previous study, soybean lunasin, a short 2S-albumin-derived peptide, had anti-inflammatory effects by inhibiting NO production in macrophages [45]. In another study, a vicilin protein suppressed NO production, with potential anti-inflammatory effects [46]. Additionally, NO production in induced inflammation cell models treated with NLL γ-conglutin protein decreased NO levels in an induced inflammatory state, showing how NLL γ-conglutin can ameliorate inflammation by lowering NO production [14].

**Figure 7:**
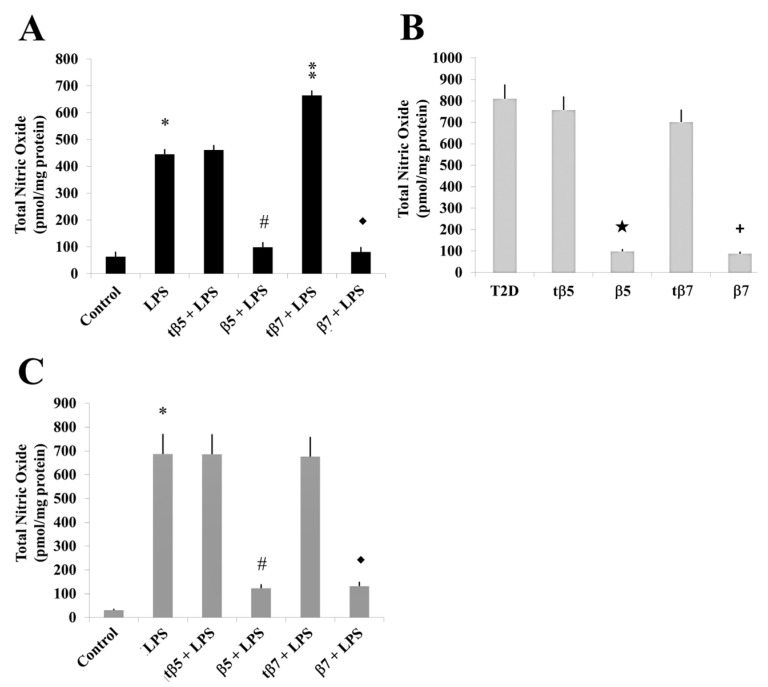
Effect of β5 and β7 conglutins on nitric oxide production. Culture cells of HepG2 (**A**), T2D (**B**), and healthy subjects (**C**). Each group of cells was incubated for 24 h in the presence of LPS, LPS+tβ5, LPS+β5, LPS+tβ7, or LPS+β7. * *p* ˂ 0.05 LPS vs. control; ** *p* ˂ 0.05 LPS+tβ5 or tβ7 vs. LPS; # *p* ˂ 0.05 LPS+β5 vs. LPS; ◆ *p* ˂ 0.05 LPS+β7 vs. LPS. ★ *p* ˂ 0.05 β5 vs. T2D and + *p* ˂ 0.05 β7 vs. T2D in T2D cell cultures. Data represent the mean ± SD of three independent experiments.

In the present study, we measured the total amount of NO, including nitrite/nitrate contents, from cultured cell supernatants of HepG2 model cells, T2D patients, and control subjects (Figure 7), and statistically analyzed the data. The total NO in LPS inflammation-induced cell cultures significantly increased to 450 and 805 nmol/min/mL (* *p* ˂ 0.05 LPS vs. control) in HepG2 cells compared to LPS-induced inflammation and healthy subjects’ culture cells, respectively, and 694 nmol/min/mL in T2D culture cells.

The NO levels significantly declined in the β5 and β7 conglutin protein treatments to 98 and 85 nmol/min/mL in HepG2 model culture cells (# *p* ˂ 0.05 LPS+β5 vs. LPS; ◆ *p* ˂ 0.05 LPS+β7 vs. LPS, respectively) (Figure 7A), 100 and 96 nmol/min/mL for T2D cells (Figure 7B), and 117 and 123 nmol/min/mL for healthy subject culture cells (Figure 7C), respectively. No significant differences (*p* < 0.05) in NO production occurred with tβ5 or tβ7 relative to the LPS inflammatory-induced states.

Thus, we can confirm that complete forms of NLL β5 and β7 conglutins regulate NO production as an additional pathway of inflammation amelioration in cells.

## 3. Materials and Methods

### 3.1. Construction of Expression Plasmids

β5 and β7 conglutins (Uniprot accession number F5B8W3 and F5B8W5, respectively) were obtained using their genetic construction (GenScript) and overexpression in the pET28b plasmid (Novogen), according to the protocol of Jimenez-Lopez et al. (2016) [21].

### 3.2. β5 and β7 Conglutin Protein Expression and Purification

β-conglutin protein isoforms were expressed in Rosetta™ 2(DE3) pLysS Singles™ Competent Cells (Novagen) following the induction protocol of Jimenez-Lopez et al. (2016) [21] with minor modifications. Both recombinant conglutin isoforms were purified using bacterial pellets rinsed with phosphate-buffered saline (PBS), pH 7.5, flash frozen in liquid nitrogen, and stored at −80 °C until further use.

Protein purification from bacterial pellets was performed using nickel affinity chromatography in Ni-NTA spin columns (Qiagen) that interact with histidine (6xHis) tags at the C-terminus of the recombinant β-conglutin proteins. After elution of 6xHis-tagged proteins by increasing the imidazole concentration gradient (15–380 mM), 2.5 mL fractions were collected.

Chromatographic fractions were analyzed using SDS–PAGE to determine the fractions containing recombinant proteins. Those showing a single band corresponding to the expected molecular weight were pooled and dialyzed five times against 100 mM Tris-HCl (pH 7.5) and 150 mM NaCl to eliminate the imidazole reagent, with the protein concentrated using a 30 kDa Amicon centrifuge filter (millipore).

The purity of the protein samples was >95% (Figure 1), with typical yields of ~17–35 mg/mL. Purified β-conglutin protein concentration was estimated using Bradford Protein Assay Kit (Thermo Fisher) and bovine serum albumin as a standard.

### 3.3. Conglutin Isoforms Antibody Production

A peptide commonly present in β5 and β7 conglutins (Nt–VDEGEGNYELVGIR–Ct) and the other five β-conglutin isoforms (β1–β4 and β6) with antigenic characteristics was synthesized to immunize experimental animals (rabbits) to produce a polyclonal antiserum. The antiserum was subsequently purified using affinity chromatography, with its concentration determined by ELISA (Agrisera) [13].

### 3.4. HepG2 Cell Culture and Treatment

HepG2 cells obtained from CIC-UGR [85011430 (batch CB No 2440)] were grown in poly-L-lysine-coated 75 cm^2^ flasks (∼2.0–2.5 × 10^6^ cells/mL) in Dulbecco’s modified Eagle’s medium (DMEM) supplemented with 2 mM glutamine and 10% heat-inactivated fetal bovine serum in the presence of 5% CO_2_/95% air at 37 °C.

Cells were routinely subcultured and used for all experiments in the exponential growth phase. Cells were cultivated as monolayers and detached by trypsinization (washed twice with PBS (Sigma) and treated with 0.25% tryp-EDTA (Lonza) for 10 min in 5% CO_2_/95% air at 37 °C in a humidified atmosphere), with the reaction neutralized using culture medium.

To neutralize the trypsinization step, culture medium was used to collect the cells immediately after centrifugation at 1000× *g* for 5 min, before washing with PBS. Cell viability was assessed using a Countess II FL Automated Cell Counter (Thermo Fisher). Cells were cultured to 80% confluence and treated with LPS (1 μg/mL) for 24 h, and individually with purified β5, β7, tβ5 or tβ7 (10 μg of each purified conglutin) for 24 h alone or combined with LPS. There were three experimental replicates for each experiment. After the treatments, cells were harvested for further analysis.

### 3.5. PBMC Isolation and Culture

Peripheral blood mononuclear cells (PBMC) were isolated from heparinized blood by density-gradient centrifugation on histopaque 1077 (Sigma), washed three times in Hanks’ balanced salt solution (Life Technologies), and resuspended in a complete medium comprising RPMI 1640 (Sigma) supplemented with penicillin, streptomycin, and L-glutamine (100 U/mL, 100 μg/mL, and 2 mM, respectively) (Sigma).

Participants fasted for 12 h before blood collection (water only, no food intake). Venous blood was drawn into lithium–heparin tubes (BD Vacutainer System). Within 3 h, whole unseparated blood was diluted 1:3 with DMEM and HEPES 2.4% (Invitrogen) and agitated gently in 50 mL tubes (Greiner Bio-one); 1 mL aliquots were seeded in 24-well plates (VWR International) and cultured for 24 h at 37 °C and 5% CO2. From each blood draw, we performed triplicate incubations in parallel with positive and negative controls—separate cultures that each included complete (β5 and β7) and truncated (tβ5 and tβ7) forms. Purified β-conglutin, respectively, LPS and PBS were used for all experiments. Blood cultures were removed from each well and centrifuged at 700× *g* for 5 min at 20 °C. The supernatants were aliquoted and stored at −20 °C until further analysis.

### 3.6. Participant Information

Fourteen T2D patients and the same number of healthy control subjects were recruited for this study from the coverage area of the Pedro Martínez Hospital (A.G.S. North East Granada, Spain).

The study was developed according to the international declarations in research ethics:

(1) Ethical of the World Medicine Association, http://www.wma.net (accessed on 18 April 2023). The Declaration of Helsinki (Edinburgh 2000).

(2) Council of Europe Convention for the Protection of Human Rights and Dignity of the Human Being with Regard to the Application of Biology and Medicine (Oviedo 1997), http://www.coe.int (accessed on 18 April 2023).

(3) UNESCO’s Universal Declaration on Bioethics and Human Rights, http://www.unesco.org (accessed on 18 April 2023).

(4) The Spanish research and ethics laws based on the CIOMS/WHO International Ethical Guidelines for Biomedical Research Involving Human Subjects, http://www.cioms.ch (accessed on 18 April 2023).

The healthy control and T2D groups included patients screened and diagnosed according to the American Diabetes Association guidelines with the following characteristics (minimum and maximum in brackets):

(A) Body mass index (kg/m^2^): healthy control subjects 24.7 (23.5, 26.3); T2D 33.8 (28.2, 45.3); (B) Blood pressure (mmHg): control 12.5/8.2 (11/7, 12.8); T2D 15.6/9.2 (14.6/8.3, 15.8/9.7); (C) Heart rate (bpm): control 68 (64, 74); T2D 85 (80, 94); (D) Fasting plasma glucose (mg/dL): control 81 (78, 90); T2D 159 (138, 183); (E) Glycated hemoglobin (HbA1c %): control 5.3 (5.1, 5.6); T2D 6.8 (6.1, 7.5).

Significant differences were found for all parameters between healthy controls and T2D subjects using analysis of variance and associated *p*-values (<0.01). This study excluded patients diagnosed with T2D that were receiving treatment for T2D.

### 3.7. Cell Viability Assay

Cell counts and viability were checked using a Countess II FL Automated Cell Counter (Thermo Fisher) at the beginning and end of each experiment using representative wells. Cell viability was monitored as >95%.

Cell viability was assessed using the MTT method [47]. Briefly, HepG2 and isolated PBMC cells were seeded in 96-well plates in the culture medium, where 103 cells were placed in 100 μL medium per well in 200 μL DMEM with FBS and antibiotics. After overnight incubation, cells were treated with LPS alone, or 10 μg each of the purified β5 and β7 conglutin protein (β5, β7 and tβ5, tβ7 forms, respectively) replicated three times. After the treatments, the cells were thoroughly washed with PBS (five times) to avoid any interference of the phenolic compounds with the MTT. Afterwards, 200 μL free red-phenol DMEM containing 1 mg/mL MTT was added, and the cells were incubated for 3 h. Viable cells convert MTT into formazone crystals (purple color), which were solubilized with 200 μL DMSO, and the absorbance measured at test (570 nm) and reference (690 nm) wavelengths using a microplate reader (Bio-Rad).

### 3.8. SDS–PAGE Protein Separation and Immunoblotting

Purified β-conglutin proteins were separated by SDS-PAGE, using 15 μg of each complete (β5 and β7) and truncated (tβ5 and tβ7) form. After electrophoresis, the proteins were visualized in gel with Coomassie Brilliant Blue for transfer to a PVDF membrane. The membranes were blocked for 2 h in 5% (*w*/*v*) non-fat dry milk in Tris-buffered saline (TBS) buffer, pH 7.4. Immunodetection of β-conglutin proteins was carried out by incubation with a primary polyclonal antiserum developed in this study (see above), diluted 1:1000 in TBS buffer containing blocking solution and 0.5% Tween-20. A secondary antibody HRP-conjugated was diluted to 1:3500 in TBS buffer and 0.5% Tween-20 for 2 h, followed by three washing steps of 15 min each with TBS containing 0.5% Tween-20. Protein bands were visualized using a chemiluminescence kit (Bio-Rad) according to the manufacturer’s instructions and the LI-COR C-DiGit Chemiluminescence Western Blot Scanner.

### 3.9. Polymerase Chain Reaction (PCR) Array for Inflammatory Response

Real-time quantitative PCR technology was used to assay TNF-α, IL-1β, and iNOS mRNA expression from each experimental group. Total RNA was extracted from HepG2 cells and isolated PBMC of T2D and control subjects using an RNeasy Mini Kit (Qiagen). Two μg of total RNA was converted to complementary DNA (cDNA) using a High-Capacity cDNA Archive Kit (Applied Biosystems). cDNA was prepared, diluted, and subjected to real-time PCR and then amplified using a TaqMan technology LightCycler 480 quantitative PCR System (Roche) for gene expression assays. Primers and probes were the same as those from commercially available TaqMan Gene Expression Assays [TNFα: Hs01555410_m1, IL-1β: Hs01075529_m1, iNOS: Hs00174128_m1, respectively]. Quantitative real-time PCR (qPCR) was performed using a Light Cycler^®^ 480 (Roche), with samples run as duplicates for 40 cycles: 1 cycle of 95 °C for 10 min, 40 cycles of 95 °C for 15 s, and 40 cycles of 60 °C for 1 min. Cycle thresholds were measured, with the relative expression of genes calculated by comparing Ct values. Differences in gene expression were evaluated by calculating the fold-change in expression levels based on at least a 2-fold up or down change relative to the control group. All gene expression levels were normalized to the housekeeping gene β beta (Assay ID: Hs99999903_m1, Applied Biosystems), which did not vary significantly between the study groups.

### 3.10. ELISA Assays for Cytokines, TNF-α, and Inflammatory Pathway Quantification

After cell culture and individual β5, β7, tβ5, and tβ7 conglutin protein challenges, the cells were counted and plated in six-well plates (106 cells per well), with two wells per group. All experiments were performed in triplicate to confirm data and reduce sample variation.

After 24 h incubation, the treated culture media was removed, and the cells were washed with 4 °C PBS. The plates were kept on ice during protein extraction to avoid denaturing the cytokines. Each well was filled with 100 μL buffer (150 mM sodium chloride, 1.0% NP-40, 50 mM Tris pH 8), supplemented with 1 μL protease inhibitor (Sigma), which remained in contact with the cells for 15 s. The cells were then vigorously scraped from the bottom of the wells, transferred to microcentrifuge tubes, and centrifuged at 12,500× *g* for 15 min at 4 °C. After centrifugation, the supernatant was collected, and 1:4 diluted for the ELISA sets for IL-1β, IL-2, IL-6, IL-8, IL12, IL-17, IL-27, TNF-α, INFγ, MPO, S-TNF-R1, S-TNF-R2, and TWEAK proteins (Diaclone) following the manufacturer’s instructions.

### 3.11. Nitric Oxide Detection

Cell supernatants of cultured cells from control subjects, T2D patients, and HepG2, were used to measure the total amount of NO production following the instructions of a commercial assay kit ab65328 (Abcam).

### 3.12. Antioxidant Enzymatic Activity Assays

The cell cultures were prepared, with the growing media removed after 24 h of incubation in the treated culture individually with β5, β7, tβ5, and tβ7, respectively, and cells washed with PBS at 4 °C. Cells from the different challenges with the different β-conglutin protein forms were collected and used to assess enzymatic activities (SOD, CAT, and GSH) (Canvax), following manufacturer’s instructions.

### 3.13. Statistical Analysis

The ELISA, NO determination, and antioxidant enzymatic activity results were evaluated using one-way ANOVA followed by Games-Howell or Tukey’s HSD post hoc tests (depending on whether variances were homogeneous). Statistical analyses were conducted at *p* < 0.05 using SPSS Statistics 27 software (IBM). Values were expressed as means ± S.E.M. of the three individual experiments.

### 3.14. β-Conglutin Proteins Structural Modeling

β5 and β7 NLL conglutin sequences (Uniprot accession numbers F5B8W3 and F5B8W5, respectively) were used to search characteristic patterns and functional (biologically meaningful) motifs using the PROSITE database, http://prosite.expasy.org/ (accessed on 18 April 2023). Additional domain architecture analyses of these proteins were performed with Pfam v25.0, http://pfam.sanger.ac.uk/ (accessed on 18 April 2023); SMART v6.0, http://smart.embl-heidelberg.de/ (accessed on 18 April 2023); Conserved Domain Database v3.02, CDART (Conserved Domain Architecture Retrieval Tool) and CD-Search tools, http://www.ncbi.nlm.nih.gov/Structure/cdd/cdd.shtml/ (accessed on 18 April 2023); InterPRO v35.0, http://www.ebi.ac.uk/interpro/ (accessed on 18 April 2023); ProDom release 2010.1, http://prodom.prabi.fr/prodom/current/html/home.php/ (accessed on 18 April 2023); CATH v3.4, http://www.cathdb.info/ (accessed on 18 April 2023); Superfamily v1.75, http://supfam.cs.bris.ac.uk/SUPERFAMILY/ (accessed on 18 April 2023); PIRSF, http://pir.georgetown.edu/pirwww/dbinfo/pirsf.shtml/ (accessed on 18 April 2023); and functional search using PANTHER http://www.pantherdb.org/ (accessed on 18 April 2023).

Structural modeling of both complete (β5 and β7) and truncated (tβ5 and tβ7) conglutin isoforms was performed by I-Tasser (a fold recognition) that works in three main steps (1) retrieving template proteins of similar folds from the PDB library, (2) excising continuous fragments from the PDB templates and reassembling into full-length models; (3) performing the fragment assembly simulation from the LOMETS templates and the PDB structures using TM-align to guide the simulations (https://zhanggroup.org/I-TASSER/about.html). An initial structural model was analyzed for recognition of errors in the 3D structure using ProSA prosa.services.came.sbg.ac.at/prosa.php (accessed on 18 April 2023) and for an overall quality estimation of the model with QMEAN swissmodel.expasy.org/qmean/cgi/index.cgi (accessed on 18 April 2023). Final β5 and β7 structures were subjected to energy minimization with GROMOS96 force field energy implemented in DeepView/Swiss-PDBViewer v3.7, spdbv.vitalit.ch, to improve van der Waals contacts and correct the model’s stereochemistry. The model’s final quality was assessed with QMEAN, stereology with PROCHECK, www.ebi.ac.uk/thornton-srv/software/PROCHECK (accessed on 18 April 2023); and ProSA, prosa.services.came.sbg.ac.at/prosa.php (accessed on 18 April 2023); and protein energy with ANOLEA, protein.bio.puc.cl/cardex/servers/anolea (accessed on 18 April 2023). The Ramachandran plot statistics for the models were calculated to show the number of protein residues in the favored regions.

Evolutionary conservational analysis was performed using the ConSurf server (consurf.tau.ac.il) by generating evolutionary-related conservation scores to identify functional regions in the proteins. Functional and structural key residues in the β-conglutin sequences were confirmed using the ConSeq server.

## 4. Conclusions

In this study, individual treatments with complete and truncated forms of NLL β-conglutin proteins (β5 and β7), applied to LPS-stimulated in vitro (HepG2) model culture cells and ex vivo (isolate PBMC from blood samples of T2D-diagnosed patients and healthy control subjects) assays, decreased mRNA expression of key pro-inflammatory molecules in T2D, including IL-1β, IL-2, IL-6, IL-8, IL-12, IL-17, IL-27, INF-γ, MOP, S-TNF-R1/-R2, TWEAK, TNF-α, and iNOS gene expression and NO production. In addition, protein levels decreased significantly for the above-mentioned pro-inflammatory mediators. Moreover, we demonstrated the importance of the mobile arm structural domain of β5 and β7 conglutin proteins in the underlying molecular mechanisms promoting anti-inflammatory actions in cells, with no comparative effects found for their counterpart forms, tβ5 and tβ7.

This study identified NLL β5 and β7 as newly discovered anti-inflammatory proteins that might play a key role as functional food components for preventive and therapeutic nutraceutical molecules of disease-related inflammation.

## Figures and Tables

**Table 1:** Changes in protein levels of pro-inflammatory cytokines and other mediators. Pro-inflammatory cytokines and other mediators measured in HepG2, in T2D and in healthy control subjects’ cell culture, respectively. Numbers represent pg/mL of proteins measured by ELISA. Positive values mean gene up-regulation. HepG2 cell culture: * *p* < 0.05 vs. control, # *p* < 0.05 vs. LPS, ♦ *p* <0.05 vs. LPS; No differences for LPS vs. LPS+tβ5 or tβ7. T2D cell culture: ◊ *p* < 0.05 vs. T2D; No differences for T2D vs. tβ5 or tβ7. Healthy control subjects’ cell culture: * *p* < 0.05 vs. control, # *p* < 0.05 vs. LPS, ♦ *p* < 0.05 vs. LPS; No differences for LPS vs. LPS+tβ5 or tβ7.

**pg/mL**	**HepG2 Control**	**LPS**	**LPS+tβ5 ^1^**	**LPS+β5**	**LPS+tβ7^1^**	**LPS+β7**
	**Mean ± SEM**	**Mean ± SEM**	**Mean ± SEM**	**Mean ± SEM**	**Mean ± SEM**	**Mean ± SEM**
IL-17	14.30 ± 0.06	140.00 ± 0.02 *	153.00 ± 0.05	11.20 ± 0.05 ^#^	156.00 ± 0.04	18.30 ± 0.01 ^♦^
IL-12	9.27 ± 0.02	241.00 ± 0.05 *	243.00 ± 0.02	76.00 ± 0.04 ^#^	271.00 ± 0.09	82.00 ± 0.02 ^♦^
IL-27	11.20 ± 0.03	198.00 ± 0.05 *	192.00 ± 0.01	87.00 ± 0.02 ^#^	176.00 ± 0.05	100.00 ± 0.04 ^♦^
IL-6	23.50 ± 0.05	287.00 ± 0.04 *	301.00 ± 0.01	178.00 ± 0.05 ^#^	262.00 ± 0.01	177.00 ± 0.06 ^♦^
IL-2	13.20 ± 0.04	56.00 ± 0.05 *	49.00 ± 0.01	32.30 ± 0.05 ^#^	52.00 ± 0.03	21.30. ± 0.05 ^♦^
IL-8	8.30 ± 0.02	79.00 ± 0.03 *	82.00 ± 0.01	41.00 ± 0.03 ^#^	80.00 ± 0.05	32.00 ± 0.01 ^♦^
MPO	9.30 ± 0.01	321.00 ± 0.10 *	439.00 ± 0.02	173.00 ± 0.05 ^#^	501.00 ± 0.03	178.00 ± 0.06 ^♦^
INFγ	44.90 ± 0.01	1907.00 ± 0.10 *	1947.00 ± 0.01	653.00 ± 0.02 ^#^	1883.00 ± 0.05	631.00 ± 0.02 ^♦^
S-TNF-R1	812.00 ± 0.01	2091.00 ± 0.06 *	1982.00 ± 0.04	932.00 ± 0.04 ^#^	1922.00 ± 0.02	921.00 ± 0.05 ^♦^
S-TNF-R2	712.00 ± 0.03	2931.00 ± 0.07 *	2786.00 ± 0.05	1023.00 ± 0.02 ^#^	2891.00 ± 0.05	1234.00 ± 0.05 ^♦^
TWEAK	87.00 ± 0.02	638.00 ± 0.06 *	676.00 ± 0.05	213.00 ± 0.01 ^#^	711.00 ± 0.03	345.00 ± 0.01 ^♦^
pg/mL	T2D	tβ5 ^1^	β5		tβ7 ^1^	β7
	Mean ± SEM	Mean ± SEM	Mean ± SEM		Mean ± SEM	Mean ± SEM
IL-17	150.30 ± 0.03	153.00 ± 0.01	11.20 ± 0.02 ^◊^		156.00 ± 0.03	18.30 ± 0.01 ^◊^
IL-12	219.27 ± 0.03	240.32 ± 0.05	76.31 ± 0.02 ^◊^		231.30 ± 0.01	81.32 ± 0.04 ^◊^
IL-27	43.20 ± 0.03	47.40 ± 0.03	23.50 ± 0.02 ^◊^		46.30 ± 0.05	26.70 ± 0.02 ^◊^
IL-6	65.50 ± 0.01	64.30 ± 0.02	36.40 ± 0.02 ^◊^		70.10 ± 0.05	41.40 ± 0.09 ^◊^
IL-2	43.00 ± 0.01	42.00 ± 0.01	22.80 ± 0.02 ^◊^		48.70 ± 0.01	29.80 ± 0.05 ^◊^
IL-8	67.00 ± 0.05	67.30 ± 0.03	36.80 ± 0.02 ^◊^		63.90 ± 0.03	35.80 ± 0.05 ^◊^
MPO	121.00 ± 0.01	119.30 ± 0.04	54.80 ± 0.02 ^◊^		128.60 ± 0.06	56.40 ± 0.06 ^◊^
INFγ	231.90 ± 0.05	242.30 ± 0.03	108.30 ± 0.02 ^◊^		227.30 ± 0.09	96.30 ± 0.02 ^◊^
S-TNF-R1	1843.00 ± 0.01	1797.30 ± 0.1	646.30 ± 0.02 ^◊^		1811.10 ± 0.09	701.30 ± 0.02 ^◊^
S-TNF-R2	1615.00 ± 0.05	1573.40 ± 0.20	936.32 ± 0.02 ^◊^		1678.30 ± 0.01	927.34 ± 0.05 ^◊^
TWEAK	198.00 ± 0.01	201.30 ± 0.04	76.30 ± 0.02 ^◊^		189.30 ± 0.09	67.90 ± 0.30 ^◊^
pg/mL	Healthy Control	LPS	LPS+tβ5 ^1^	LPS+β5	LPS+tβ7 ^1^	LPS+β7
	Mean ± SEM	Mean ± SEM	Mean ± SEM	Mean ± SEM	Mean ± SEM	Mean ± SEM
IL-17	1.66 ± 0.02	143.00 ± 0.02 *	153.00 ± 0.04	11.20 ± 0.01 ^#^	156.00 ± 0.03	18.30 ± 0.01 ^♦^
IL-12	27.76 ± 0.05	241.00 ± 0.03 *	243.00 ± 0.09	76.00 ± 0.05 ^#^	251.00 ± 0.05	81.30 ± 0.01 ^♦^
IL-27	256.70 ± 0.08	473.30 ± 0.04 *	421.20 ± 0.06	238.40 ± 0.09 ^#^	491.30 ± 0.05	298.40 ± 0.05 ^♦^
IL-6	16.80 ± 0.03	198.30 ± 0.05 *	187.40 ± 0.10	57.40 ± 0.04 ^#^	194.30 ± 0.04	67.40 ± 0.06 ^♦^
IL-2	14.50 ± 0.02	79.40 ± 0.09 *	78.40 ± 0.01	34.60 ± 0.06 ^#^	81.30 ± 0.05	36.60 ± 0.05 ^♦^
IL-8	9.40 ± 0.06	145.60 ± 0.09 *	152.40 ± 0.02	24.50 ± 0.04 ^#^	148.40 ± 0.10	32.40 ± 0.08 ^♦^
MPO	5.80 ± 0.02	349.40 ± 0.09 *	374.50 ± 0.04	163.40 ± 0.02 ^#^	356.40 ± 0.02	174.40 ± 0.03 ^♦^
INFγ	37.50 ± 0.04	284.40 ± 0.01 *	278.40 ± 0.08	87.40 ± 0.04 ^#^	291.40 ± 0.09	79.40 ± 0.05 ^♦^
S-TNF-R1	801.30 ± 0.09	1489.40 ± 0.10 *	1456.80 ± 0.01	783.40 ± 0.01 ^#^	1581.10 ± 0.08	900.00 ± 0.02 ^♦^
S-TNF-R2	765.30 ± 0.05	1932.30 ± 0.05 *	1911.30 ± 0.05	609.90 ± 0.01 ^#^	1900.30 ± 0.04	1239.90 ± 0.06 ^♦^
TWEAK	86.30 ± 0.06	684.30 ± 0.06 *	711.20 ± 0.03	211.30 ± 0.03 ^#^	765.40 ± 0.09	309.30 ± 0.05 ^♦^

^1^ No statistically significant differences to the compared group (LPS or T2D).

## Data Availability

Not applicable.

## Supplementary Materials

The following supporting information can be downloaded at: https://www.mdpi.com/article/10.3390/ijms24087676/s1.
