# Supplementary material for: β-Conglutins’ Unique Mobile Arm Is a Key Structural Domain Involved in Molecular Nutraceutical Properties of Narrow-Leafed Lupin (Lupinus angustifolius L.)
Source: Int J Mol Sci. 2023 Apr 21;24(8):7676. doi: 10.3390/ijms24087676 (PMC10143110; doi:10.3390/ijms24087676)
Supplement: Supplementary file 1 [file ijms-24-07676-s001.zip › Table S2.pdf]

**Table S2. Viability (%) assessment of human peripheral blood mononuclear cells (PBMC).**

Viability was measured by MTT assay for isolated PBMC culture, conglutins β5 or β7, LPS, LPS + β5 or β7, LPS + t β5 or tβ7 during 24 hours.

Treatments including conglutin β5 or β7 (normal or truncated forms) were added at 10 μg, and LPS at 1 μg. Data represent mean ± SD from three independent experiments.

| **Samples** | **Viability** | |
|---|---|---|
| | **T2D patients** | **Healthy subjects** |
| LPS | 97.5 ± 3.5 | 98.3 ± 2.0 |
| Conglutin β5 | 102.5 ± 2.3 | 100.5 ± 1.5 |
| Conglutin tβ5 | 105.0 ± 4.4 | 102.0 ± 3.3 |
| Conglutin β7 | 99.0 ± 2.2 | 98.0 ± 2.2 |
| Conglutin tβ7 | 98.9 ± 2.5 | 98.4 ± 2.0 |
| Conglutin β5 + LPS | 98.3 ± 2.1 | 97.8 ± 1.8 |
| Conglutin tβ5 + LPS | 97.4 ± 3.5 | 99.2 ± 1.5 |
| Conglutin β7 + LPS | 96.5 ± 5.6 | 101.5 ± 4.3 |
| Conglutin tβ7 + LPS | 101.3 ± 2.4 | 100.7 ± 1.4 |
