# Supplementary material for: β-Conglutins’ Unique Mobile Arm Is a Key Structural Domain Involved in Molecular Nutraceutical Properties of Narrow-Leafed Lupin (Lupinus angustifolius L.)
Source: Int J Mol Sci. 2023 Apr 21;24(8):7676. doi: 10.3390/ijms24087676 (PMC10143110; doi:10.3390/ijms24087676)
Supplement: Supplementary file 1 [file ijms-24-07676-s001.zip › Figure-S2.pdf]

A

```
F5B8W4_β6  MIKMRVRFPTLVLLLGIVFLMAVSIGIAYGEKNVIKNHERPQEREQEERDPRQQPRPHHQEEQEREHRREEERDREPSRGRRESEESREEEREQRREPRREREQEQQPQHGRREEEEE--
F5B8W2_β4  MIKMRVRFPTLVLLLGIVFLMAVSIGIAYGEKNVIKNHERPQEREQEERDPRQQPRPHHQEEQEREHRREEERDREPSRGRSESEESREEEREQRREPRREREQEQQPQHGRREEEEE--
F5B8W1_β3  MAKMRVRFPTLVLLLGIVFLMAVSIGIAYGEKNVLKNHERPQEREQEERDPRQQPRPHHQEEQEREHRRE-------------SEESQEEEREQRREPRREREQEQQPQHGRREEEEE--
F5B8W0_β2  MANMRVKFPTLVLLLGIVFLMAVSIGIAYGEKNAIKNHERPQEREQEERDPRQQPRPRHQEEQEREHGREEERNREPSRGRSESEESREEEREQRREPSRGREQEQQPQHGRREEEEE--
F5B8W5_β5  MAKMRVRFPMLVLLLGVVFLLAVSIGIAYGEKDVIKNPERPEERQEEERDPRQPPRSRQQEEQEREHRREKERDREPSRGRSESKQSQEEEERERRKEHDREREQEQQPQYGRRHEEEEKG
F5B8W3_β7  MARMRVRFPTLVLLLGILFLMAVSIGIAYGEKDVIKNHERPGEREHEERDPRQQPRPRKQEEQEREHRREEEHDRDPSRGRRESEERQEEERERRREPCREREQEQQPQHGRREEEEE--
F5B8V9_β1  MAKMRVRLPMLILLLGVVFLLAASIGIAYGEKDFTKNP--PKEREEEEHEPRQQPRPRQQEEQEREHRREEKHDGEPSRGRSQSEESQEEEHERRREHHREREQEQQPRPQRRQEEEEE
Consensus  * .***::* *:****::**:*.*********:  **   * **:.**::*** **.::******* **           *:: :***:*:*:*  * *******:  **.****

F5B8W4_β6  -----WQPRRQRPQSRREEREQEQGSSSSSRRQSAYERREQREEREQE--------------------------------QEQGSRSDSRRQRNPYYFSSERFQTLYRNRNGQIRVLER
F5B8W2_β4  -----WQPRRQRPQSRREEREQEQGSSSSSRRQSGYERRE---EREQE--------------------------------QEQGSRSDSRRQRNPYYFSSERFQTLYRNRNGQIRVLER
F5B8W1_β3  -----WQPRRQRPQSRREEREQEQGSSSSSRRQSGYERREQREEREQE--------------------------------QEQGSRSDSRRQRNPYYFSSERFQTLYRNRNGQIRVLER
F5B8W0_β2  -----WQPRRQRPQSRREEREQEQGSSSSSGRQSGYERREQREEREQQ--------------------------------QEQDSRSESRRQRNPYYFSYERFQTLYKNRNGQIRVLER
F5B8W5_β5  EEEEEGQARRQRPQRREEREQEQGSSSESRRQSGDERRHRHEKREQREEREQEQGSSSGRQSDYGRRQRHEGREQREEREQEQGSSSESHRLRNPYYFSSERFQTRYKNKNGQIRVLER
F5B8W3_β7  --EEEWQPRRLRPQSRKEEREQEQGSSSSSRKQSGYERRQYHERREQRDEKE----------------------------KEQDSRSDSRRQRNPYHFSSERFQTRYRNRNGQIRVLER
F5B8V9_β1  EEWQPRRQRPQSRREEREREEREQEQGSSSGSQRGSGDERRQHRERRVHREERE----------------------------QEQDSRSDSRRQRNPYHFSSNRFQTYYRNRNGQIRVLER
Consensus        : *    :  .:*********** * : *. ***.    .* :.                                :**.* *:*:* ****:** :**** *:*:*********

F5B8W4_β6  FDKRTDRLENLQNYRIVEFQSKPNTLILPKHSDADYILVVLNGSATITIVNPDKRQSYNLENGDALRLPAGTTSYILNPDDNQNLRVVKLAIPINNPGNFYDFYPSSSKDQQSYFSGFSR
F5B8W2_β4  FDQRTDRLENLQNYRIVEFQSKPNTLILPKHSDADYILVVLNGSATITIVNPDKRQSYNLENGDALRLPAGTTSYILNPDDNQNLRVVKLAIPINNPGNFYDFYPSSSKDQQSYFSGFSR
F5B8W1_β3  FDQRTNRLENLQNYRIVEFQSKPNTLILPKHSDADYILVVLNGSATITIVNPDKRQSYNLENGDALRLPAGTTSYILNPDDNQNLRVVKLAIPINNPGNFYDFYPSSSKDQQSYFSGFSK
F5B8W0_β2  FDQRTNRLENLQNYRIVEFQSKPNTLILPKHSDADYILVVLNGRATITIVNPDKRQAYNLEHGDALRLPAGTTSYILNPDDNQNLRVVKLAIPINNPGNFYDFYPSSTKDQQSYFNGFSR
F5B8W5_β5  FDQRTNRLENLQNYRIVEFQSRPNTLILPKHSDADYILVVLNGRATITIVNPDKRQAYNLEYGDALRLPAGTTSYILNPDDNQDLRVVKLAIPINNPGKFYDFYPSRTKDQQSYFSGFSK
F5B8W3_β7  FDQRTNRLENLQNYRIVEFQSNPNTLILPKHSDADYILVVLNGRATITIVNPDKRQAYNLEYGDALRVPAGTTSYILNPDDNQNLRVVKLAIPINNPSNFYDFYPSSTKDQQSYFSGFSK
F5B8V9_β1  FNQRTNRLENLQNYRIIEFQSKPNTLILPKHSDADFILVVLNGRATITIVNPDKRQVYNLEQGDALRLPAGTTSYILNPDDNQNLRVAKLAIPINNPGKLYDFYPSTTKDQQSYFSGFSK
Consensus  *::**:**********:****.*************:******* ************ **** *****:**************:***.*********.::****** :*******.***:

F5B8W4_β6  NTLEATFNTRYEEIQRILLGNEDEQEDDEQRHGQEQSHQDEGVIVRVSKEQVQELRKYAQSSSRKGKPSKSGPFNLRSNKPIYSNKFGNFYEITPNRNPQAQDLDISLTFIEINEGALLL
F5B8W2_β4  NTLEATFNTRYEEIQRILLGNEDEQEDDEQRHGQEQSHQDEGVIVRVSKEQVQELRKYAQSSSRKGKPSKSGPFNLRSNKPIYSNKFGNFYEITPNRNPQAQDLDISLTFIEINEGALLL
F5B8W1_β3  NTLEATFNTRYEEIQSILLGNEDEQEDDEQWHGQEQSHQDEGVIVRVSKEQVQELRKYAQSSSRKGKPYESGPFNLRSNKPIYSNKFGNFYEITPDRNPQAQDLDISLTFIEINEGALLL
F5B8W0_β2  NTLEATFNTRYEEIQRIILGNEDGQEDEEQSRGQEQSHQDQGVIVRVSKEQIQELRKHAQSSSGKGKPSESGPFNLRSDEPIYSNKFGNFYEITPDRNPQAQDLDISLTFIEINEGGLLL
F5B8W5_β5  NTLEATFNTHYEEIQRILLGYEDEQEDEEQRREQEQSHQDEGVIVRVSKEQIQELRKHAQSSSRKGKPSESGPFNLRSNEPIYSNKFGNFYEITPDRNPQVQDLDISLIFTEISEGALLL
F5B8W3_β7  NTLEATFNTRYEEIQRILLGNEDEQEDEEQRRGQEQSYQDEGVIVRVSKEQIQELRKHAQSSSRKGKPSESGPFNLRSNESIYSNKFGNFYEITPERNPQVQDLDISLTFTEINEGALLL
F5B8V9_β1  NTLEATFNTRYEEIERVLLGDDELQENEKQRRGQEQSHQDEGVIVRVSKKQIQELRKHAQSSSGEGKPSESGPFNLRSNKPIYSNKFGNFYEITPDINPQFQDLNISLTFTEINEGALLL
Consensus  *********:****: ::** :: **:::* : ****:**:********:*:*****:***** :*** :*********::.**************: *** ***:*** * **.**.***

F5B8W4_β6  PHYNSKAIFVVLVDEGEGNYELVGIRDQQRQQDEQE---------VRRYSARLSEGDIFVIPAGHPISINASSNFRLLGFGINADENQRNFLAGFEDNVIRQLDREVKGLTFPGFAEDVE
F5B8W2_β4  PHYNSKAIFVVLVDEGEGNYELVGIRDQQRQQDEQE---------VRRYSARLSEGDIFVIPAGHPISINASSNLRLLGFGINADENQRNFLAGSEDNVIRQLDTEVKGLTFPGSTEDVE
F5B8W1_β3  PHYNSKAIFVVVVDEGEGNYELVGIRDQQRQQDEQE---------VRRYSARLSEGDIFVIPAGHPISINASSNLRLLGFGINADENQRNFLAGSEDNVIRQLDREVKGLIFPGSAEDVE
F5B8W0_β2  PHYNSKAIFVVVVDEGEGNYELVGIRDQERQQDEQEQE-------VRRYNAKLSEGDIFVIPAGHPISINASSNLRLLGFGINADENQRNFLAGSEDNVIRQLDKEVKQLTFPGSVEDVE
F5B8W5_β5  PHYNSKAIFVIVVDEGEGNYELVGIRNQQRQQDEQEVEE------VRSYNARLSEGDILVIPAGHPLSINASSNLRLLGFGINADENQRNFLAGSEDNVIRQLDREVKELIFPGSAEDVE
F5B8W3_β7  PHYNSKAIFIVVVDEGEGNYELVGIRDQQRQQDEQEEEEE----EVRRYSARLSEGDIFVIPAGYPISVNASSNLRLLGFGINANENQRNFLAGSEDNVISQLDREVKELTFPGSAQDVE
F5B8V9_β1  PHYNSKAIFIVVVDEGEGNYELVGIRDQQRQQDEQEEEYEQGEEEVRRYSDKLSKGDVFIIPAGHPLSINASSNLRLLGFGINANENQRNFLAGSEDNVIKQLDREVKELTFPGSIEDVE
Consensus  *********:::**************:*:*******         ** *. :**:**:::****:*:*:*****:*********:*********  ***** *** *** * ***   :***

F5B8W4_β6  RLIKNQQQSYFANAQPQQQQQ-REREGRHGRRGHIFSILSTLY-------
F5B8W2_β4  RLIKNQQQSYFANAQPQQQQQ-REREGRRGRRGHISSILSTLY-------
F5B8W1_β3  RLIKNQQQSYFANAQPQQQQQ-REREGRHGRRGHISSILSTLY-------
F5B8W0_β2  RLIKNQQQSYFANAQPQQQQQ-REKEGRRGRRGLSFPFRSLFTKLLSTIM
F5B8W5_β5  RLIRNQQQSYFANAQPQQQQQQREKEGRRGRRGPISSILSALY-------
F5B8W3_β7  RLIKNQQQSYFANAQPQQKQQ-REKEGRRGRRSLISSILSTLY-------
F5B8V9_β1  RLIKNQQQSYFANAQPQQQQQ-REKEGRRGRRGPISSILNALY-------
Consensus  ***:**************:** **:***:***.   .: . :
```

B

```
F5B8V9_β1   EQDSRSDSRRQRNPYHFSSNRFQTYYRNRNGQIRVLERFNQRTNRLENLQNYRIIEFQSKPNTLILPKHSDADFILVVLNGRATITIVNPDKRQVYNLEQGDALRLPAGTTSYILNPDDN
F5B8W3_β7   EQDSRSDSRRQRNPYHFSSERFQTRYRNRNGQIRVLERFDQRTNRLENLQNYRIVEFQSNPNTLILPKHSDADYILVVLNGRATITIVNPDKRQAYNLEYGDALRVPAGTTSYILNPDDN
F5B8W2_β4   EQGSRSDSRRQRNPYYFSSERFQTLYRNRNGQIRVLERFDQRTDRLENLQNYRIVEFQSKPNTLILPKHSDADYILVVLNGSATITIVNPDKRQSYNLENGDALRLPAGTTSYILNPDDN
F5B8W4_β6   EQGSRSDSRRQRNPYYFSSERFQTLYRNRNGQIRVLERFDKRTDRLENLQNYRIVEFQSKPNTLILPKHSDADYILVVLNGSATITIVNPDKRQSYNLENGDALRLPAGTTSYILNPDDN
F5B8W1_β3   EQGSRSDSRRQRNPYYFSSERFQTLYRNRNGQIRVLERFDQRTNRLENLQNYRIVEFQSKPNTLILPKHSDADYILVVLNGSATITIVNPDKRQSYNLENGDALRLPAGTTSYILNPDDN
F5B8W2_β2   EQDSRSESRRQRNPYYFSYERFQTLYKNRNGQIRVLERFDQRTNRLENLQNYRIVEFQSKPNTLILPKHSDADYILVVLNGRATITIVNPDKRQAYNLEHGDALRLPAGTTSYILNPDDN
F5B8W5_β5   EQGSSSESHRLRNPYYFSSERFQTRYKNKNGQIRVLERFDQRTNRLENLQNYRIVEFQSRPNTLILPKHSDADYILVVLNGRATITIVNPDKRQAYNLEYGDALRLPAGTTSYILNPDDN
Consensus   **.* *:*:* ****:** :**** *:*:**********::**:**********:****.*************:******* ************ **** *****:**************

F5B8V9_β1   QNLRVAKLAIPINNPGKLYDFYPSTTKDQQSYFSGFSKNTLEATFNTRYEEIERVLLGDDELQENEKQRRGQEQSHQDEGVIVRVSKKQIQELRKHAQSSSGEGKPSESGPFNLRSNKPI
F5B8W3_β7   QNLRVVKLAIPINNPSNFYDFYPSSTKDQQSYFSGFSKNTLEATFNTRYEEIQRILLGNEDEQEDEEQRRGQEQSYQDEGVIVRVSKEQIQELRKHAQSSSRKGKPSESGPFNLRSNESI
F5B8W2_β4   QNLRVVKLAIPINNPGNFYDFYPSSSKDQQSYFSGFSRNTLEATFNTRYEEIQRILLGNEDEQEDDEQRHGQEQSHQDEGVIVRVSKEQVQELRKYAQSSSRKGKPSKSGPFNLRSNKPI
F5B8W4_β6   QNLRVVKLAIPINNPGNFYDFYPSSSKDQQSYFSGFSRNTLEATFNTRYEEIQRILLGNEDEQEDDEQRHGQEQSHQDEGVIVRVSKEQVQELRKYAQSSSRKGKPSKSGPFNLRSNKPI
F5B8W1_β3   QNLRVVKLAIPINNPGNFYDFYPSSSKDQQSYFSGFSKNTLEATFNTRYEEIQSILLGNEDEQEDDEQWHGQEQSHQDEGVIVRVSKEQVQELRKYAQSSSRKGKPYESGPFNLRSNKPI
F5B8W2_β2   QNLRVVKLAIPINNPGNFYDFYPSSTKDQQSYFNGFSRNTLEATFNTRYEEIQRIILGNEDGQEDEEQSRGQEQSHQDQGVIVRVSKEQIQELRKHAQSSSGKGKPSESGPFNLRSDEPI
F5B8W5_β5   QDLRVVKLAIPINNPGKFYDFYPSRTKDQQSYFSGFSKNTLEATFNTHYEEIQRILLGYEDEQEDEEQRREQEQSHQDEGVIVRVSKEQIQELRKHAQSSSRKGKPSESGPFNLRSNEPI
Consensus   *:***.*********.::****** :*******.***:*********:****: ::** :: **:::* : ****:**:********:*:*****:***** :*** :********::.*

F5B8V9_β1   YSNKFGNFYEITPDINPQFQDLNISLTFTEINEGALLLPHYNSKAIFIVVVDEGEGNYELVGIRDQQRQQDEQEEEYEQGEEEVRRYSDKLSKGDVFIIPAGHPLSINASSNLRLLGFGI
F5B8W3_β7   YSNKFGNFYEITPERNPQVQDLDISLTFTEINEGALLLPHYNSKAIFIVVVDEGEGNYELVGIRDQQRQQDEQEE----EEEEVRRYSARLSEGDIFVIPAGYPISVNASSNLRLLGFGI
F5B8W2_β4   YSNKFGNFYEITPNRNPQAQDLDISLTFIEINEGALLLPHYNSKAIFVVLVDEGEGNYELVGIRDQQRQQDEQE---------VRRYSARLSEGDIFVIPAGHPISINASSNLRLLGFGI
F5B8W4_β6   YSNKFGNFYEITPNRNPQAQDLDISLTFIEINEGALLLPHYNSKAIFVVLVDEGEGNYELVGIRDQQRQQDEQE---------VRRYSARLSEGDIFVIPAGHPISINASSNFRLLGFGI
F5B8W1_β3   YSNKFGNFYEITPDRNPQAQDLDISLTFIEINEGALLLPHYNSKAIFVVVVDEGEGNYELVGIRDQQRQQDEQE---------VRRYSARLSEGDIFVIPAGHPISINASSNLRLLGFGI
F5B8W2_β2   YSNKFGNFYEITPDRNPQAQDLDISLTFIEINEGGLLLPHYNSKAIFVVVVDEGEGNYELVGIRDQERQQDEQEQ------EEVRRYNAKLSEGDIFVIPAGHPISINASSNLRLLGFGI
F5B8W5_β5   YSNKFGNFYEITPDRNPQVQDLDISLIFTEISEGALLLPHYNSKAIFVIVVDEGEGNYELVGIRNQQRQQDEQEV------EEVRSYNARLSEGDILVIPAGHPLSINASSNLRLLGFGI
Consensus   *************: *** ***:*** * **.**.************:::********** ****:*:*******         ** *. :**:**:::****:*:*:*****:*******

F5B8V9_β1   NANENQRNFLAGSEDNVIKQLDREVKELTFPGSIEDVERLIKNQQQSYFANAQPQQQQQ-REKEGRRGRRGPISSILNALY-------
F5B8W3_β7   NANENQRNFLAGSEDNVISQLDREVKELTFPGSAQDVERLIKNQQQSYFANAQPQQKQQ-REKEGRRGRRSLISSILSTLY-------
F5B8W2_β4   NADENQRNFLAGSEDNVIRQLDTEVKGLTFPGSTEDVERLIKNQQQSYFANAQPQQQQQ-REREGRRGRRGHISSILSTLY-------
F5B8W4_β6   NADENQRNFLAGFEDNVIRQLDREVKGLTFPGFAEDVERLIKNQQQSYFANAQPQQQQQ-REREGRHGRRGHIFSILSTLY-------
F5B8W1_β3   NADENQRNFLAGSEDNVIRQLDREVKGLIFPGSAEDVERLIKNQQQSYFANAQPQQQQQ-REREGRHGRRGHISSILSTLY-------
F5B8W2_β2   NADENQRNFLAGSEDNVIRQLDKEVKQLTFPGSVEDVERLIKNQQQSYFANAQPQQQQQ-REKEGRRGRRGLSFPFRSLFTKLLSTIM
F5B8W5_β5   NADENQRNFLAGSEDNVIRQLDREVKELIFPGSAEDVERLIRNQQQSYFANAQPQQQQQQREKEGRRGRRGPISSILSALY-------
Consensus   **:********* ***** *** *** * ***  :******:**************:** **:***:***.   .: . :
```

C

```
F5B8W3_β7    MARMRVRFPTLVLLLGILFLMAVSIGIAYGEKDVIKNHERPGEREHEERDPRQQPRPRKQEEQEREHRREEEHDRDPSRGRRESEERQEEERERRREPCREREQEQQPQHGR----REEE
F5B8W3_tβ7   ------------------------------------------------------------------------------------------------------------------------
F5B8W5_β5    MAKMRVRFPMLVLLLGVVFLLAVSIGIAYGEKDVIKNPERPEERQEEERDPRQPPRSRQQEEQEREHRREKERDREPSRGRSESKQSQEEERERRKEHDREREQEQQPQYGRRHEEEEKG
F5B8W5_tβ5   ------------------------------------------------------------------------------------------------------------------------
Consensus

F5B8W3_β7    EEEEEWQPRRLRPQSRKEEREQEQGSSSSSRKQSGYERRQ-----------------------------YHERREQRDEKEKEQDSRSDSRRQRNPYHFSSERFQTRYRNRNGQIRVLER
F5B8W3_tβ7   ----------------------------------------------------------------------------------EQDSRSDSRRQRNPYHFSSERFQTRYRNRNGQIRVLER
F5B8W5_β5    EEEEEGQARRQRPQRRREEREQEQGSSSESRRQSGDERRHRHEKREQREEREQEQGSSSGRQSDYGRRQRHEGREQREEREQEQGSSSESHRLRNPYYFSSERFQTRYKNKNGQIRVLER
F5B8W5_tβ5   ----------------------------------------------------------------------------------EQGSSSESHRLRNPYYFSSERFQTRYKNKNGQIRVLER
Consensus                                                                                      **.* *:*:* ****:**********:*:*********

F5B8W3_β7    FDQRTNRLENLQNYRIVEFQSNPNTLILPKHSDADYILVVLNGRATITIVNPDKRQAYNLEYGDALRVPAGTTSYILNPDDNQNLRVVKLAIPINNPSNFYDFYPSSTKDQQSYFSGFSK
F5B8W3_tβ7   FDQRTNRLENLQNYRIVEFQSNPNTLILPKHSDADYILVVLNGRATITIVNPDKRQAYNLEYGDALRVPAGTTSYILNPDDNQNLRVVKLAIPINNPSNFYDFYPSSTKDQQSYFSGFSK
F5B8W5_β5    FDQRTNRLENLQNYRIVEFQSRPNTLILPKHSDADYILVVLNGRATITIVNPDKRQAYNLEYGDALRLPAGTTSYILNPDDNQDLRVVKLAIPINNPGKFYDFYPSRTKDQQSYFSGFSK
F5B8W5_tβ5   FDQRTNRLENLQNYRIVEFQSRPNTLILPKHSDADYILVVLNGRATITIVNPDKRQAYNLEYGDALRLPAGTTSYILNPDDNQDLRVVKLAIPINNPGKFYDFYPSRTKDQQSYFSGFSK
Consensus    ********************.**********************************************:***************:*************.:******* *************

F5B8W3_β7    NTLEATFNTRYEEIQRILLGNEDEQEDEEQRRGQEQSYQDEGVIVRVSKEQIQELRKHAQSSSRKGKPSESGPFNLRSNESIYSNKFGNFYEITPERNPQVQDLDISLTFTEINEGALLL
F5B8W3_tβ7   NTLEATFNTRYEEIQRILLGNEDEQEDEEQRRGQEQSYQDEGVIVRVSKEQIQELRKHAQSSSRKGKPSESGPFNLRSNESIYSNKFGNFYEITPERNPQVQDLDISLTFTEINEGALLL
F5B8W5_β5    NTLEATFNTHYEEIQRILLGYEDEQEDEEQRREQEQSHQDEGVIVRVSKEQIQELRKHAQSSSRKGKPSESGPFNLRSNEPIYSNKFGNFYEITPDRNPQVQDLDISLIFTEISEGALLL
F5B8W5_tβ5   NTLEATFNTHYEEIQRILLGYEDEQEDEEQRREQEQSHQDEGVIVRVSKEQIQELRKHAQSSSRKGKPSESGPFNLRSNEPIYSNKFGNFYEITPDRNPQVQDLDISLIFTEISEGALLL
Consensus    *********:********** *********** ****:******************************************.**************:************ ****.******

F5B8W3_β7    PHYNSKAIFIVVVDEGEGNYELVGIRDQQRQQDEQEEEEEEVRRYSARLSEGDIFVIPAGYPISVNASSNLRLLGFGINANENQRNFLAGSEDNVISQLDREVKELTFPGSAQDVERLIK
F5B8W3_tβ7   PHYNSKAIFIVVVDEGEGNYELVGIRDQQRQQDEQEEEEEEVRRYSARLSEGDIFVIPAGYPISVNASSNLRLLGFGINANENQRNFLAGSEDNVISQLDREVKELTFPGSAQDVERLIK
F5B8W5_β5    PHYNSKAIFVIVVDEGEGNYELVGIRNQQRQQDEQE--VEEVRSYNARLSEGDILVIPAGHPLSINASSNLRLLGFGINADENQRNFLAGSEDNVIRQLDREVKELIFPGSAEDVERLIR
F5B8W5_tβ5   PHYNSKAIFVIVVDEGEGNYELVGIRNQQRQQDEQE--VEEVRSYNARLSEGDILVIPAGHPLSINASSNLRLLGFGINADENQRNFLAGSEDNVIRQLDREVKELIFPGSAEDVERLIR
Consensus    *********::***************:*********   **** *.********:*****:*:*:***************:*************** ********* *****:******:

F5B8W3_β7    NQQQSYFANAQPQQKQQ-REKEGRRGRRSLISSILSTLY
F5B8W3_tβ7   NQQQSYFANAQPQQKQQ-REKEGRRGRRSLISSILSTLY
F5B8W5_β5    NQQQSYFANAQPQQQQQQREKEGRRGRRGPISSILSALY
F5B8W5_tβ5   NQQQSYFANAQPQQQQQQREKEGRRGRRGPISSILSALY
Consensus    **************:** **********. ******:**
```

D

```
F5B8W5_β5    MAKMRVRFPMLVLLLGVVFLLAVSIGIAYGEKDVIKNPERPEERQEEERDPRQPPRSRQQEEQEREHRREKERDREPSRGRSESKQSQEEERERRKEHDREREQEQQPQYGRRHEEEEKG
F5B8W3_β7    MARMRVRFPTLVLLLGILFLMAVSIGIAYGEKDVIKNHERPGEREHEERDPRQQPRPRKQEEQEREHRREEEHDRDPSRGRRESEERQEEERERRREPCREREQEQQPQHGRREEEEE--
Consensus    **:****** ******::**:**************** *** **:.******* **.*:*:**********:*:**:***** **:: ********:*  **********:***.****

F5B8W5_β5    EEEEEGQARRQRPQRRREEREQEQGSSSESRRQSGDERRHRHEKREQREEREQ
F5B8W3_β7    --EEEWQPRRLRPQSRKEEREQEQGSSSSSRKQSGYERRQYHERREQRDEKEK
Consensus      *** *.** *** *:***********.**:*** ***: **:****:*:*:
```
