# Supplementary material for: β-Conglutins’ Unique Mobile Arm Is a Key Structural Domain Involved in Molecular Nutraceutical Properties of Narrow-Leafed Lupin (Lupinus angustifolius L.)
Source: Int J Mol Sci. 2023 Apr 21;24(8):7676. doi: 10.3390/ijms24087676 (PMC10143110; doi:10.3390/ijms24087676)
Supplement: Supplementary file 1 [file ijms-24-07676-s001.zip › Table S1.pdf]

Table S1. Viability (%) assessment of HEPG2 cell culture.

Viability was measured by MTT assay for HEPG2 cell culture, conglutins β5 or β7, LPS, LPS + β5 or β7, LPS + t β5 or tβ7.

Treatments including conglutin β5 or β7 (normal or truncated forms) were added at 10 μg, and LPS at 1 μg. Data represent mean ± SD from three independent experiments.

Samples	Viability
LPS	99.0 ± 1.5
Conglutin β5	98.4 ± 3.7
Conglutin tβ5	97.0 ± 5.3
Conglutin β7	99.0 ± 2.6
Conglutin tβ7	100.0 ± 4.3
Conglutin β5 + LPS	95.4± 4.7
Conglutin tβ5 + LPS	97.2 ± 5.5
Conglutin β7 + LPS	101.4± 3.6
Conglutin tβ7 + LPS	102.6± 5.2
