# Supplementary material for: β-Conglutins’ Unique Mobile Arm Is a Key Structural Domain Involved in Molecular Nutraceutical Properties of Narrow-Leafed Lupin (Lupinus angustifolius L.)
Source: Int J Mol Sci. 2023 Apr 21;24(8):7676. doi: 10.3390/ijms24087676 (PMC10143110; doi:10.3390/ijms24087676)
Supplement: Supplementary file 1 [file ijms-24-07676-s001.zip › Supplementary Figure Captions.pdf]

SUPPLEMENTARY FIGURE CAPTIONS

Figure S1. β5 and β7 conglutins structural analysis.

Three-dimensional structure of β5 and β7 conglutins (Uniprot accession number F5B8W3 and F5B8W5, respectively) showing the mobile arm and globular domains. The structures are depicted as a cartoon diagram integrated by α-helices, β-sheets, and coils (red, yellow, and green, respectively). Three-dimensional structure of (A) tβ5, (C) tβ7, (B) β5, and (D) β7 conglutins depicted in (B, D) cartoon and (C, E) surface.

Figure S2. β-conglutin protein sequences analysis.

Alignment of the complete NLL conglutin proteins sequences β1 to β7 (A); Alignment of the globular domain of the NLL conglutin protein sequences β1 to β7 (B); Comparative alignment of the NLL conglutin protein sequences β5, β7, tβ5, and tβ7 (C); Comparative alignment of the mobile-arm domain of the NLL conglutin protein sequences tβ5 and tβ7 (D).
