# Supplementary figures and images for: β-Conglutins’ Unique Mobile Arm Is a Key Structural Domain Involved in Molecular Nutraceutical Properties of Narrow-Leafed Lupin (Lupinus angustifolius L.)
Source: Int J Mol Sci. 2023 Apr 21;24(8):7676. doi: 10.3390/ijms24087676 (PMC10143110; doi:10.3390/ijms24087676)

**A**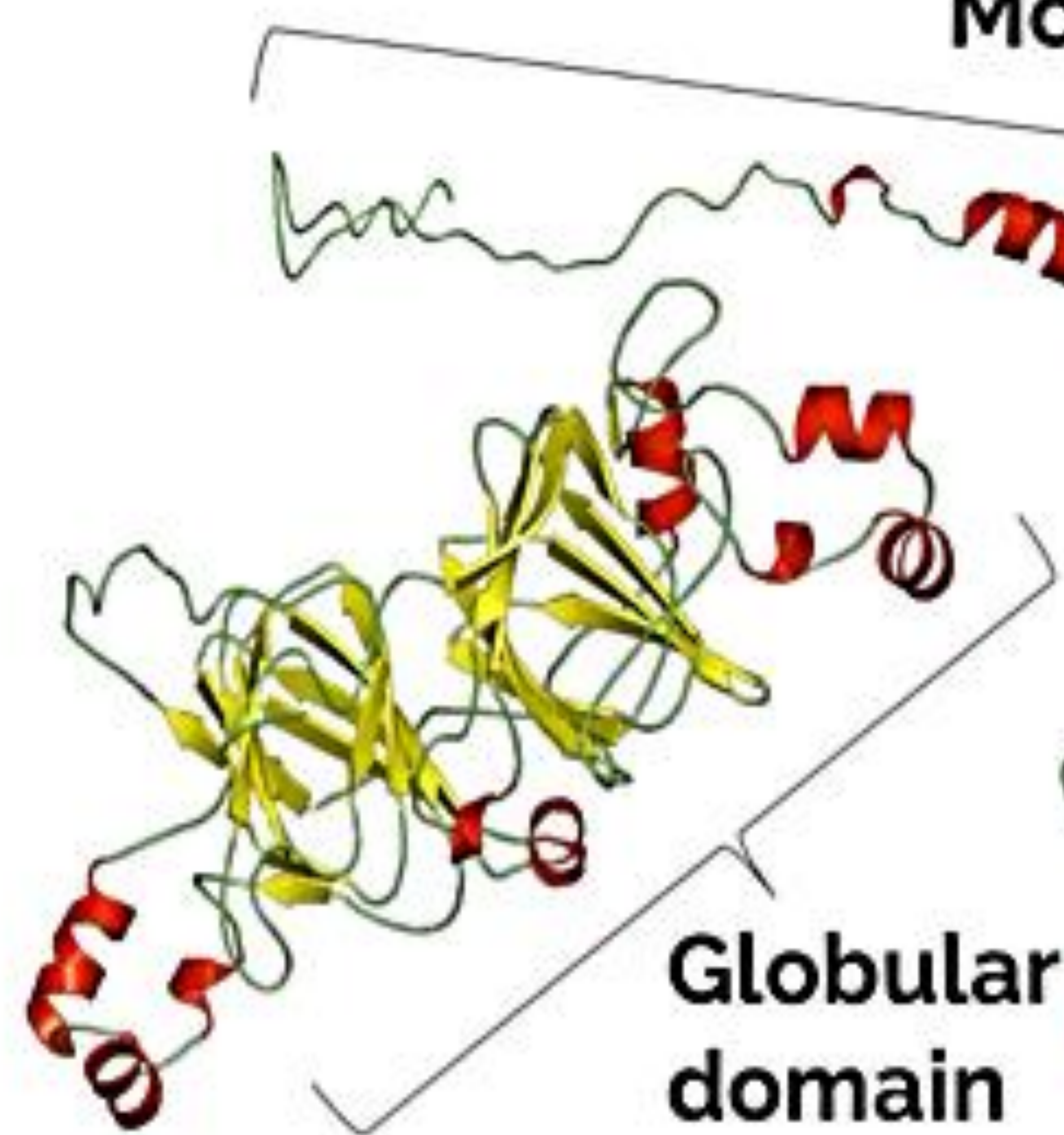**B****Mobile arm**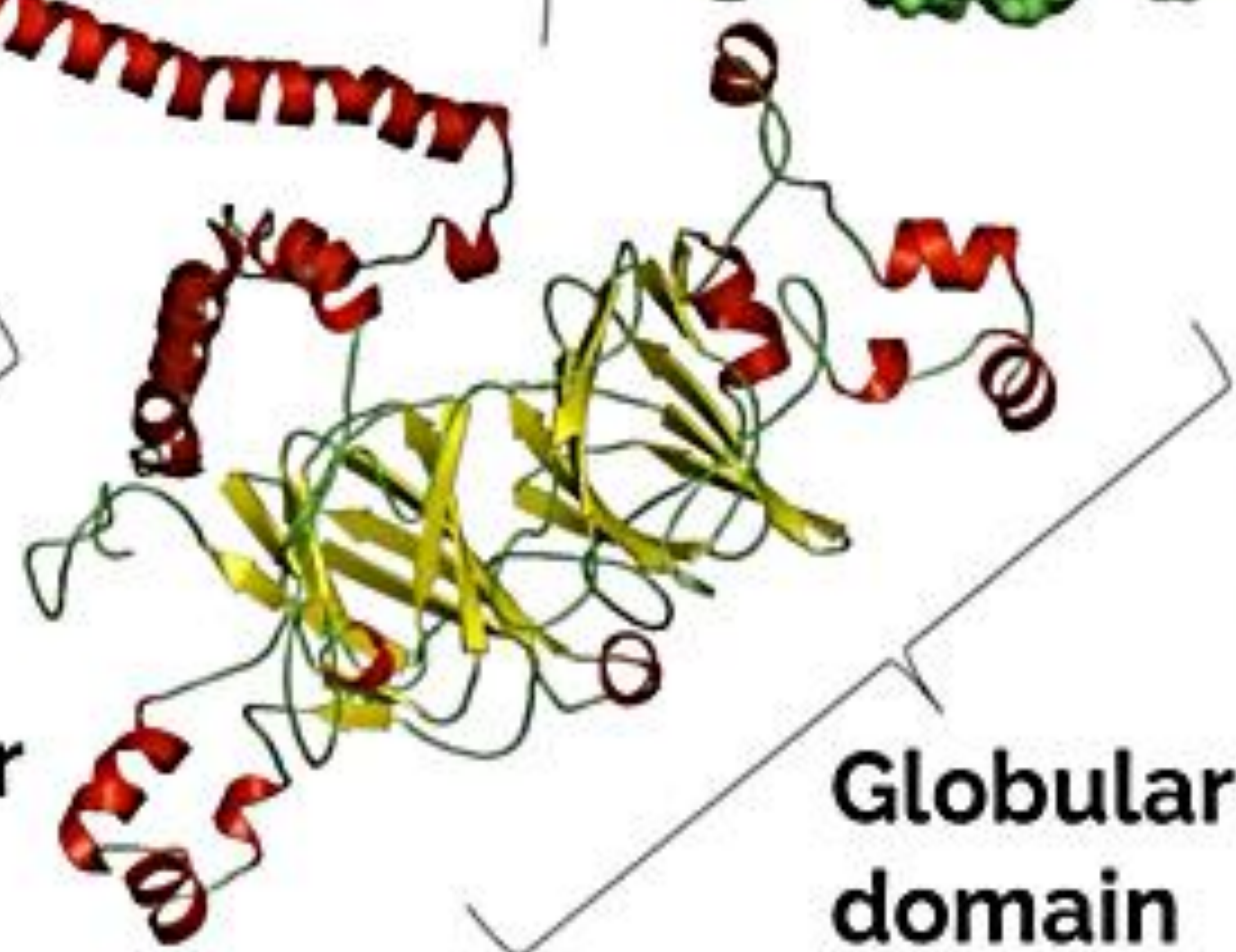**C****Mobile arm**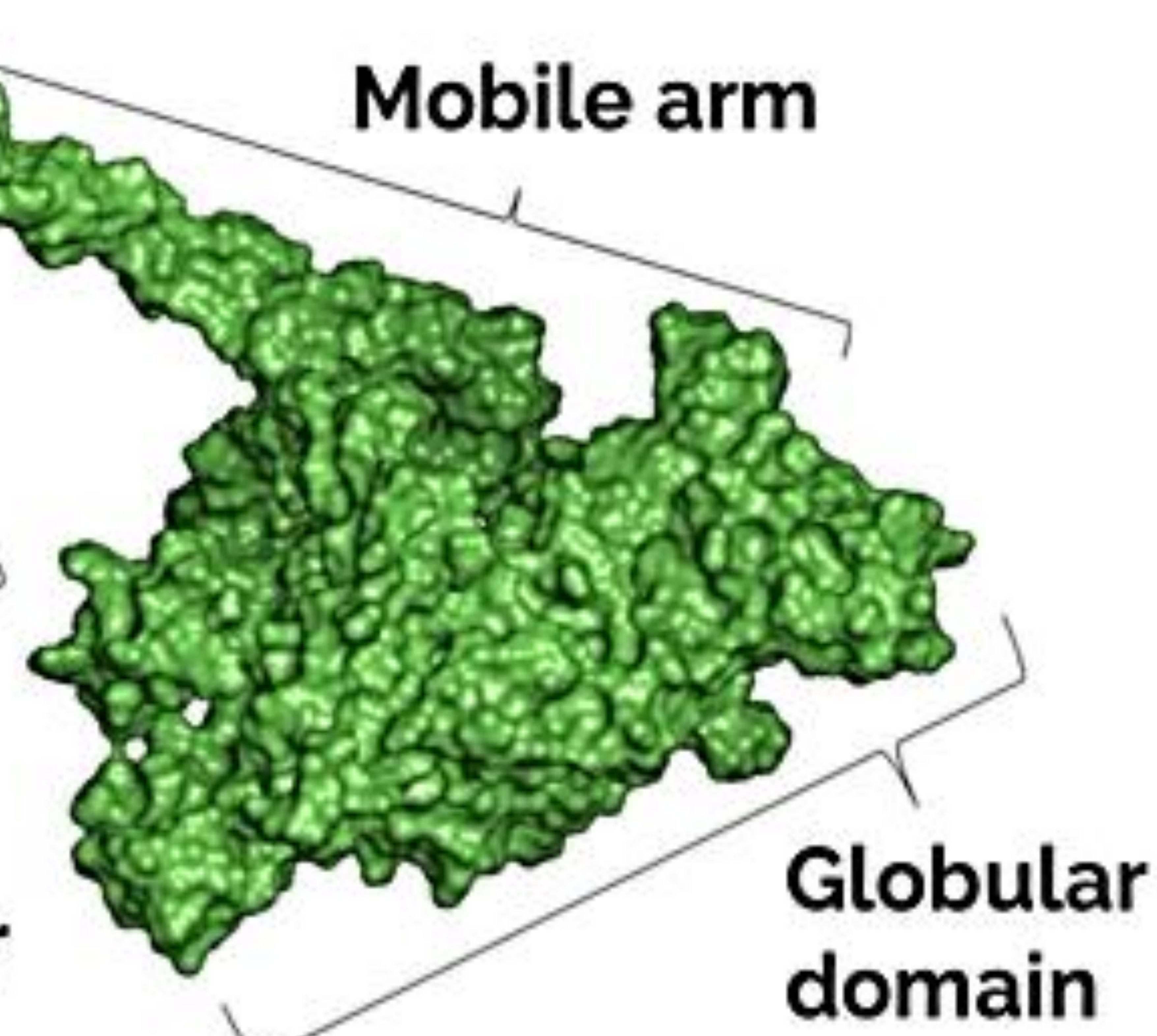**C**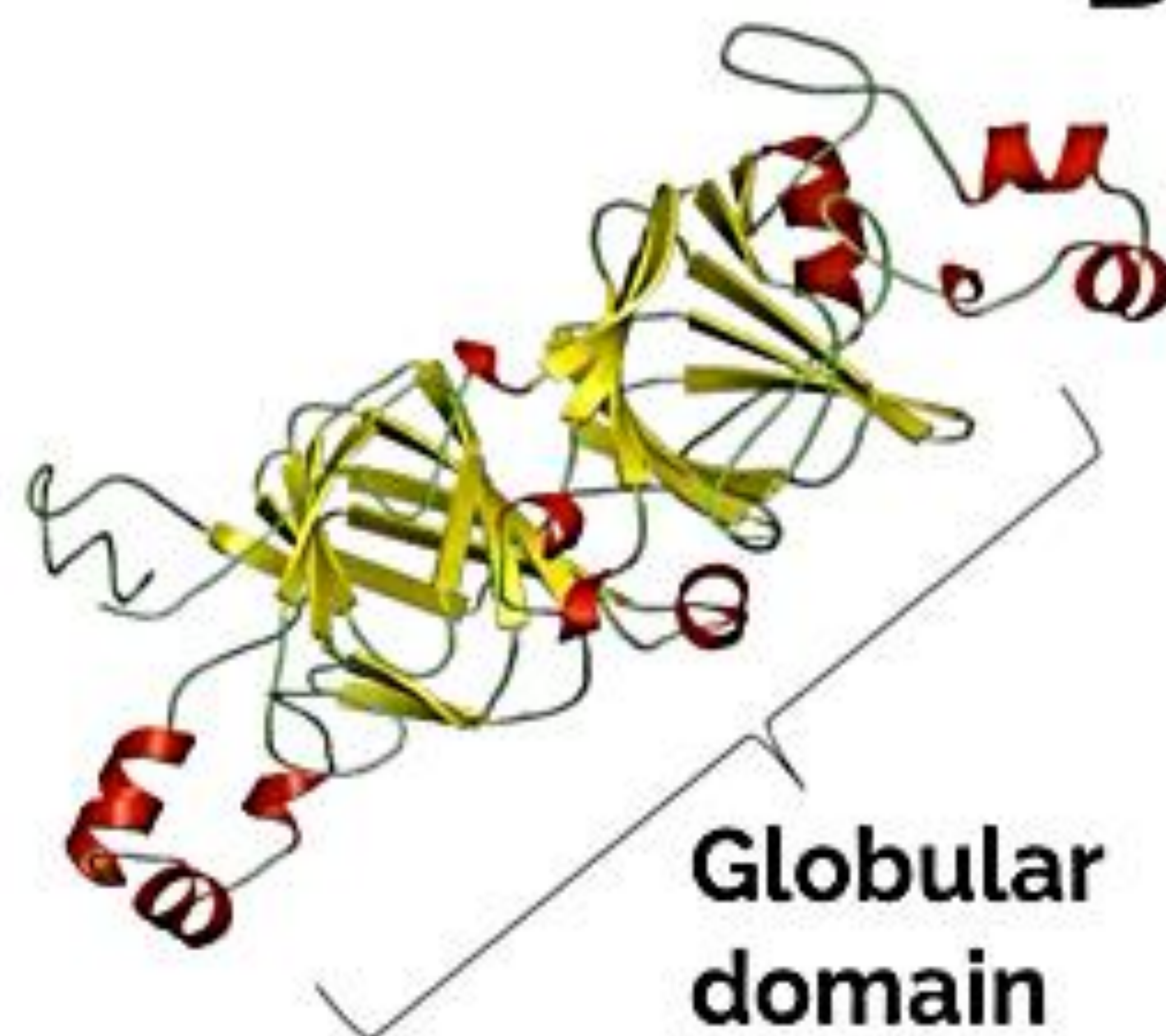**D****Mobile arm**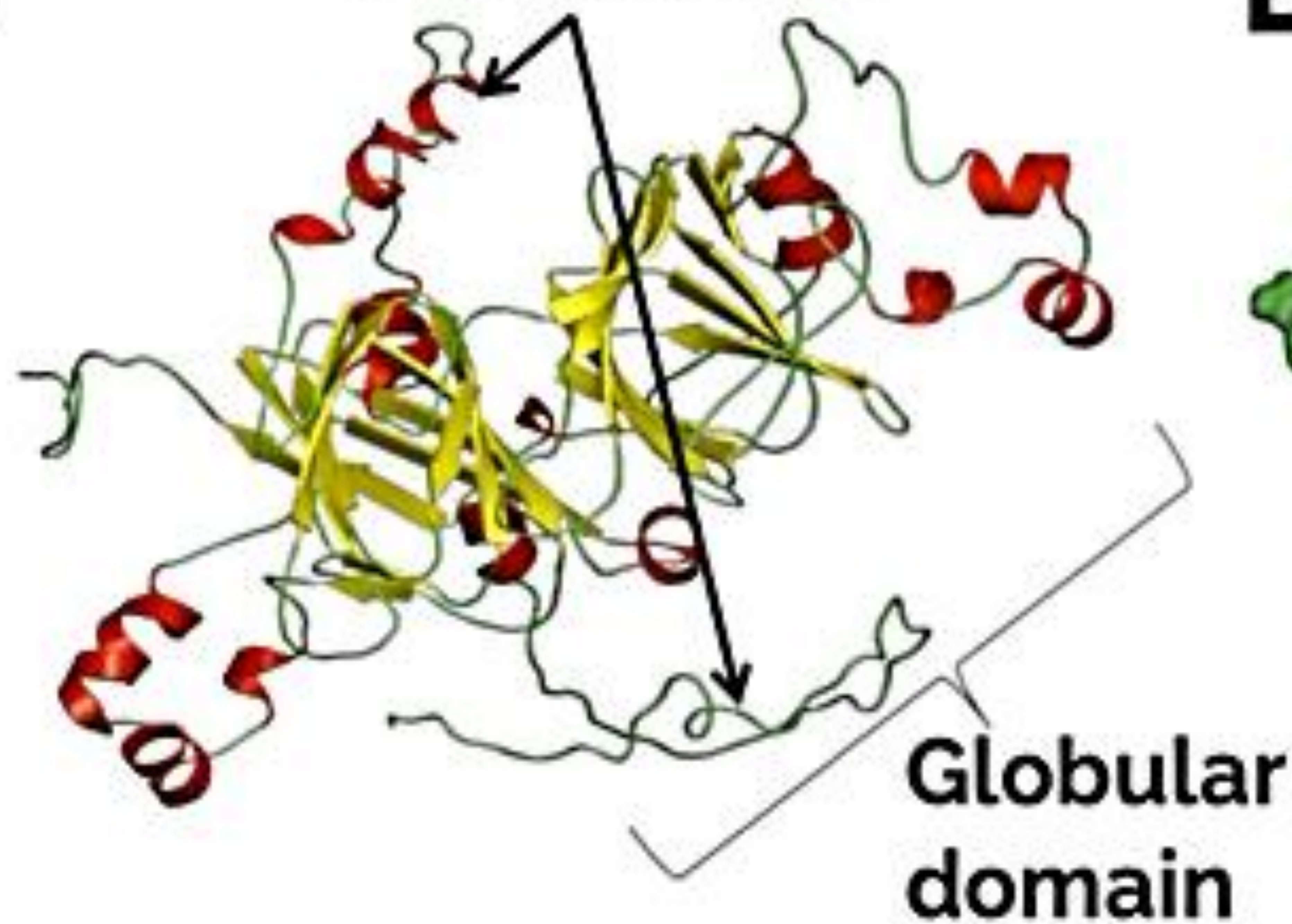**E****Mobile arm**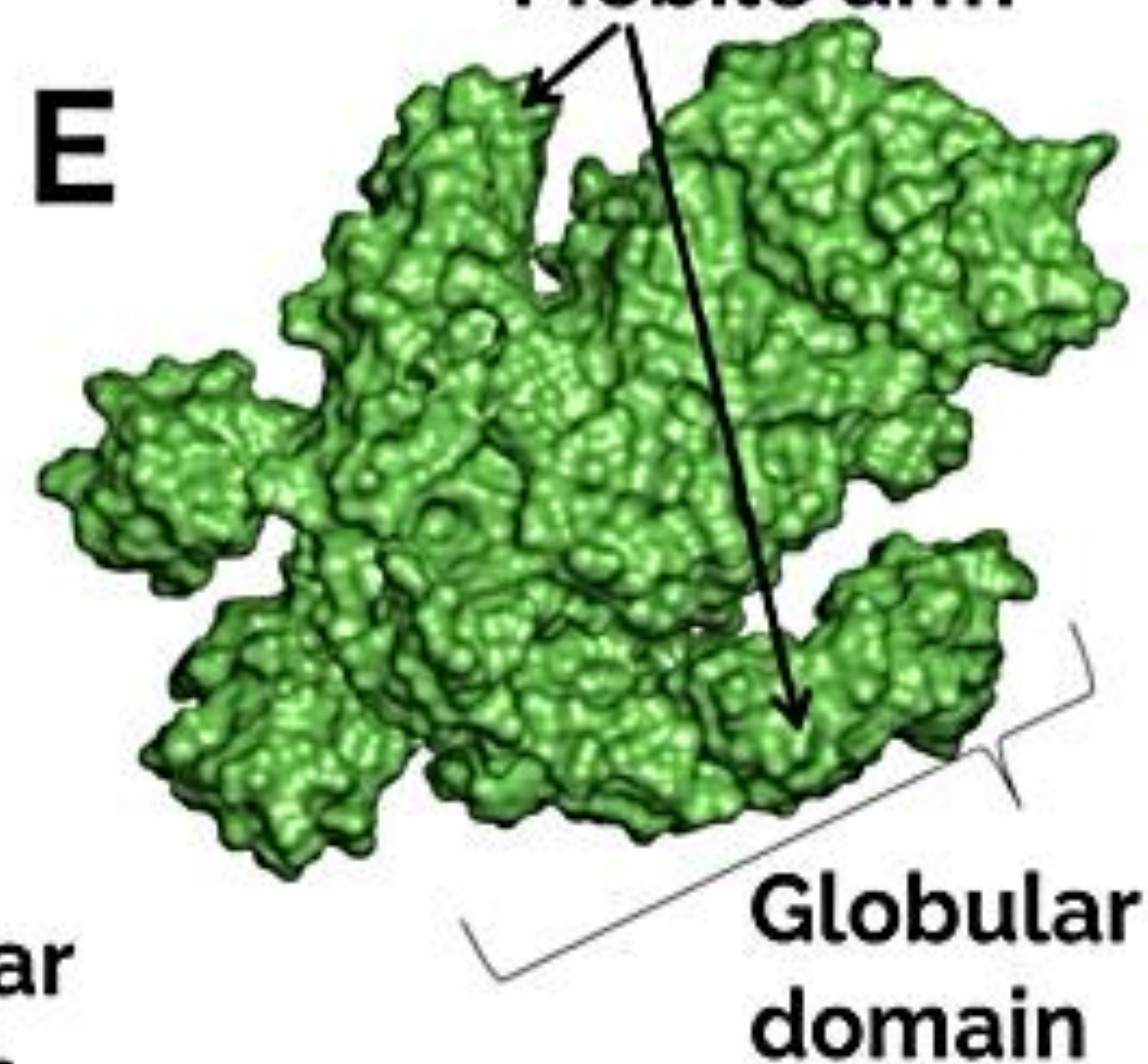

Supplement: Supplementary file 1 [file ijms-24-07676-s001.zip › Figure S1.pdf]
